# Draft genome assemblies using sequencing reads from Oxford Nanopore Technology and Illumina platforms for four species of North American *Fundulus* killifish

**DOI:** 10.1093/gigascience/giaa067

**Published:** 2020-06-18

**Authors:** Lisa K Johnson, Ruta Sahasrabudhe, James Anthony Gill, Jennifer L Roach, Lutz Froenicke, C Titus Brown, Andrew Whitehead

**Affiliations:** Department of Environmental Toxicology, University of California. 1 Shields Avenue, Davis, CA 95616, Davis, CA, USA; Department of Population Health & Reproduction, School of Veterinary Medicine, University of California. 1 Shields Avenue, Davis, CA 95616, Davis, CA, USA; DNA Technologies Core, Genome Center, University of California, 1 Shields Avenue, Davis, CA 95616; Department of Environmental Toxicology, University of California. 1 Shields Avenue, Davis, CA 95616, Davis, CA, USA; Department of Environmental Toxicology, University of California. 1 Shields Avenue, Davis, CA 95616, Davis, CA, USA; DNA Technologies Core, Genome Center, University of California, 1 Shields Avenue, Davis, CA 95616; Department of Population Health & Reproduction, School of Veterinary Medicine, University of California. 1 Shields Avenue, Davis, CA 95616, Davis, CA, USA; Department of Environmental Toxicology, University of California. 1 Shields Avenue, Davis, CA 95616, Davis, CA, USA

**Keywords:** long reads, Oxford Nanopore, killifish, genomes, genome assemblies, polish

## Abstract

**Background:**

Whole-genome sequencing data from wild-caught individuals of closely related North American killifish species (*Fundulus xenicus, Fundulus catenatus, Fundulus nottii*, and *Fundulus olivaceus*) were obtained using long-read Oxford Nanopore Technology (ONT) PromethION and short-read Illumina platforms.

**Findings:**

Draft *de novo* reference genome assemblies were generated using a combination of long and short sequencing reads. For each species, the PromethION platform was used to generate 30–45× sequence coverage, and the Illumina platform was used to generate 50–160× sequence coverage. Illumina-only assemblies were fragmented with high numbers of contigs, while ONT-only assemblies were error prone with low BUSCO scores. The highest N50 values, ranging from 0.4 to 2.7 Mb, were from assemblies generated using a combination of short- and long-read data. BUSCO scores were consistently >90% complete using the Eukaryota database.

**Conclusions:**

High-quality genomes can be obtained from a combination of using short-read Illumina data to polish assemblies generated with long-read ONT data. Draft assemblies and raw sequencing data are available for public use. We encourage use and reuse of these data for assembly benchmarking and other analyses.

## Background

Sequencing and assembling large eukaryotic genomes is challenging [1–3]. The accuracy of downstream analyses, such as selection scans, synteny analysis, and measuring gene expression, depends on high-quality reference genome assemblies [4]. Fortunately, as costs of generating whole-genome sequence data decrease, it is becoming easier for individual laboratories rather than large consortiums to generate assemblies for organisms without reference genomes [3, 5, 6]. Single-molecule long-read nucleic acid sequencing technology from Oxford Nanopore Technologies (ONT) has been commercially available since 2014 [7]. This technology has been shown to improve the contiguity of reference assemblies [8] and reveal “dark regions” that were previously camouflaging genes [9]. The lengths of the sequencing reads generated using this technology are limited only by the size of the fragments in the extracted DNA sample [10]. The promise of more complete reference assemblies is especially important for the accuracy of comparative evolutionary genomics studies because assembly fragments lead to errors in downstream synteny analyses [11], as well as single-nucleotide polymorphism calling and identification of transcript features (splice junctions and exons) for quantification.

Despite high error rates of ONT reads ∼5% [12] relative to Illumina short reads ∼0.3% [13] and the relatively recent availability of ONT data, there has been recent expansion of genome projects using this sequencing technology. Small genomes from bacteria and viruses seem to be ideal for sequencing on the ONT MinION platform [12]. The portable nature of the technology makes it appealing as a resource for teaching [14, 15], working in remote locations [16–18], and for investigating viral outbreak public health emergencies [19–21]. However, despite the demonstrated ability to achieve yields >6.5 Gb per flow cell [22], the MinION platform can be prohibitively expensive for sequencing larger eukaryotic genomes. For example, 39 flow cells yielded 91.2 Gb of sequence data (∼30× coverage) of the human genome [23]. Sequencing of the wild tomato species *Solanum pennellii* across 31 flow cells yielded 111.0 Gb (∼100× coverage) with some flow cells yielding >5 Gb [24]. By contrast, following the 2018 beta release of the ONT PromethION platform, which has a higher density of nanopore channels, 5 flow cells were used to yield >250.0 Gb (∼80× coverage) of the human genome [25]. PromethION data combined with Hi-C long-range mapping data from human samples produced a genome assembly with a scaffold N50 of 56.4 Mb [26]. While changes in pore chemistry and protocols are improving the yields from the ONT MinION, the yield from the ONT PromethION platform is larger because of the higher density of nanopore channels.

The combination of long-read sequencing data from ONT MinION and short-read sequencing data from Illumina has been used to improve the quality of reference genomes [27–30]. In one approach, short-read assembly scaffolds have been improved with the addition of long reads. The Murray cod genome (640–669 Mb in size) was improved by combining low coverage (804 Mb) of long-read ONT data from just 1 MinION flow cell with 70.6 Gb of Illumina data from both HiSeq and MiSeq; the assembly scaffold N50 increased from 33,442 bp (Illumina only) to 52,687 bp with ONT and Illumina combined [31]. The clownfish genome (791–794 Mb in size) was improved by including 8.95 Gb of ONT MinION reads; the scaffold N50 increased from 21,802 bp (Illumina only) to 401,715 bp with ONT and Illumina combined [27]. Consensus building with racon [32] and/or pilon [33] tools uses Illumina data to “polish” contigs from ONT-only assemblies. Polishing corrects single-nucleotide base differences, fills gaps, and identifies local misassemblies [33]. This approach has been shown to improve the BUSCO score from <1% with the ONT assembly alone to >95% complete after polishing with Illumina reads, with significant reduction of indels and homozygous and heterozygous single-nucleotide polymorphisms [28].

In this study, we explored whether the ONT PromethION sequencing technology could be appropriate for generating draft reference genomes for 4 species of North American killifish belonging to the *Fundulus* genus. *Fundulus* is a comparative model system for studying evolutionary divergence between marine and freshwater environments. *Fundulus* killifish are broadly distributed across North America. These small cyprinodontiform fish have evolved to occupy a wide range of osmotic niches, including marine, estuarine, and freshwater [34]. Estuarine and coastal *Fundulus* are euryhaline, insofar as they can adjust their physiologies to tolerate a very wide range of salinities. In contrast, freshwater species are stenohaline: they tolerate a much narrower range of salinities [34, 35]. Freshwater clades are derived from marine clades, and radiation into fresh water has occurred multiple times independently within the genus. This makes *Fundulus* unusual because most large clades of fishes are either exclusively marine or exclusively freshwater. Therefore, species of closely related killifish in the *Fundulus* genus serve as a unique comparative model system for understanding the genomic mechanisms that contribute to evolutionary divergence and convergence of osmoregulatory processes, which is important for understanding how species will cope with changing salinity regimes expected with climate change [36]. The Atlantic killifish*, Fundulus heteroclitus*, has been a well-described model organism for investigating physiological resilience to temperature, salinity, hypoxia, and environmental pollution [34, 37–39]. There is a reference genome available for *F. heteroclitus* [40]. However, no reference genomes exist from other *Fundulus* killifish, particularly from those occupying freshwater habitats.

**Figure 1:**
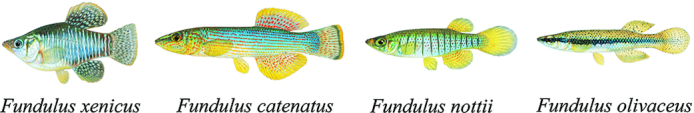
Four *Fundulus* killifish (*left to right*): the marine diamond killifish, *Fundulus xenicus*; the freshwater northern studfish, *Fundulus catenatus* (south central United States); the freshwater bayou topminnow, *Fundulus nottii*; and the freshwater blackspotted topminnow, *Fundulus olivaceus*. (drawings used with permission from the artist, Joseph R. Tomelleri).

Here, we report the collection of whole-genome sequencing data using both ONT PromethION and Illumina platforms from 4 killifish species without previously existing sequencing data (Fig. 1): *Fundulus xenicus* (NCBI:txid722643, Fishbase ID: 3166; formerly *Adinia xenica*) [41], *Fundulus catenatus* (NCBI:txid34776, Fishbase ID: 3186), *Fundulus nottii* (NCBI:txid54270, Fishbase ID: 3198), and *Fundulus olivaceus* (NCBI:txid34782, Fishbase ID: 3199). *F. xenicus* is euryhaline and occupies coastal and estuarine habitats, while the other species (*F. catenatus, F. nottii, F. olivaceus*) are stenohaline and occupy freshwater habitats.

## Methods and Results

Live field-caught individuals of each fish species were identified by field experts, shipped to University of California Davis, and kept at their native salinities in an animal holding facility maintained according to University of California IACUC standards. *F. catenatus* and *F. olivaceus* were collected from the Gasconade River, MO (latitude/longitude coordinates 37.879/−91.795 and 37.19/−92.56, respectively); *F. nottii* was collected from Walls Creek, MS (31.154433/-89.245381); and *F. xenicus* was collected from Graveline Bayou, MS (30.368756/-88.719329). High molecular weight (hmw) DNA was extracted from fresh tissue for *F. nottii* and *F. xenicus*, and from frozen tissue for *F. catenatus* and *F. olivaceus*. For *F. catenatus* and *F. olivaceus*, tissues were dissected and frozen in liquid nitrogen then stored immediately at −80°C until samples were prepared for hmw DNA extraction. With the exception of *F. olivaceus*, each assembly consisted of sequencing 1 tissue sample from 1 individual. For *F. olivaceus*, Illumina data were collected from DNA extracted from 1 individual while the ONT PromethION data were collected from another individual (frozen tissue).

### DNA extractions

**Figure 2:**
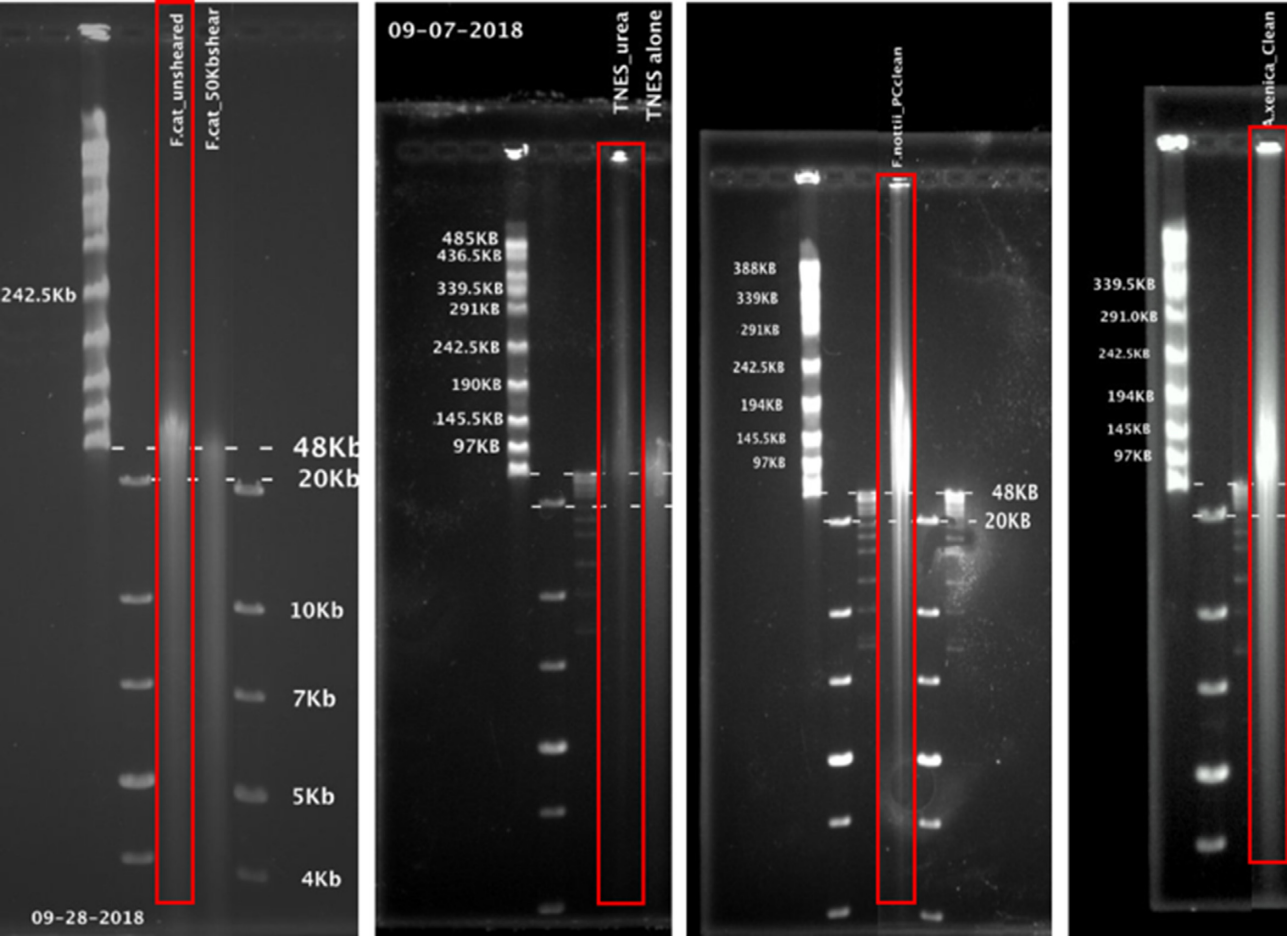
Field inversion gels with red boxes showing samples sequenced (in order from left to right: *F. catenatus* (sheared vs unsheared), *F. olivaceus, F. nottii, F. xenicus*). DNA was extracted from fresh tissues for *F. xenicus* and *F. nottii*, and from frozen tissues for *F. catenatus* and *F. olivaceus*.

Whole fish heads were used for hmw DNA extractions. Agilent's (Santa Clara, CA, USA) Genomic DNA Isolation kit (Catalog No. 200600) was used to extract DNA from fresh tissues from *F. xenicus* and *F. nottii*. For *F. catenatus* and *F. olivaceus*, 2 extraction methods were tested: (i) Tris, NaCl, EDTA, SDS, and Proteinase K followed by phenol: chloroform extraction [42] and (ii) Qiagen's (Germantown, MD, USA) Gentra Puregene Tissue Kit (Catalog No. 158667). These were both found to be similar to the Agilent kit. Precipitated DNA was difficult to re-dissolve; therefore, additional phenol: chloroform clean-up steps were added after extractions. We found that adding urea to the lysis buffer helped to precipitate the DNA pellet, making it less fragile and go into solution easier [43]. Prior to library preparation, hmw DNA from *F. nottii* and *F. olivaceus* (PromethION) was sheared to 50 kb in an effort to improve the ligation enzyme efficiency, resulting in fragments in the 50–70 kb range. Field inversion gels were used to visualize hmw DNA (Fig. 2).

### ONT sequencing

Libraries for ONT PromethION sequencing were prepared using the ligation sequencing kit (Oxford Nanopore Technologies, New York, NY, USA: SQK-LSK109) following the manufacturer's instructions. ONT PromethION sequencing data were collected from all 4 species on an alpha-beta instrument through the early release program at the University of California, Davis DNA Technologies Core facility (Davis, CA, USA). One species was sequenced per R9.4 flow cell (PRO001 and PRO002). Base-calling was done onboard the PromethION instrument using MinKnow versions 2.0–2.2 (Oxford Nanopore Technologies, New York, NY, USA). Flow cell and base-caller versions can be found in Supplemental Table 1. For the *F. xenicus* run, lambda phage (DNA CS) was spiked in as a positive control.

### Illumina sequencing

With the exception of *F. olivaceus*, each individual hmw DNA sample used for the ONT library was also used for Illumina library preparation using the Nextera Index Kit (Illumina, Inc., San Diego, CA, USA: FC-121-1012). For each of *F. catenatus, F. nottii*, and *F. xenicus*, Illumina data were multiplexed across 2 PE150 lanes on an Illumina HiSeq 4000 (Illumina HiSeq 4000, RRID:SCR_016386) and demultiplexed by Novogene (Sacramento, CA, USA). For *F. olivaceus*, PE150 Illumina NovaSeq reads from 1 flow cell (2 lanes) were graciously provided by the Texas A&M AgriLife Research Sequencing Facility (College Station, TX, USA).

## Data Description

Whole-genome sequencing data from individuals of 4 killifish species collected from ONT PromethION (Table 1) and Illumina (NovaSeq and HiSeq 4000) (Table 2) were deposited in the European Nucleotide Archive (ENA) under the study accession PRJEB29136. Deposited raw data are untrimmed and unfiltered. Reads corresponding to lambda phage were filtered from ONT PromethION data using the NanoLyse program from NanoPack (version 1.1.0 [44]). Porechop (Porechop, RRID:SCR_016967) version 0.2.3 was used to remove residual ONT adapters, and NanoFilt (NanoFilt, RRID:SCR_016966) version 2.2.0 [44] was used to filter reads with an average Phred quality score >Q5. After filtering and adapter trimming, ONT data from the PromethION ranged from 30 to 45× coverage for each species. NanoPlot (version 1.10.0 [44]) was used for visualization of ONT read qualities.

**Table 1:** ONT data collected from each species

Species	Bases called (Gb)	Coverage (×)	Mean read length	Reads N50	Q>5 bases called (Gb)	Q>5 mean read length	ONT signal accession	ONT FASTQ accession
*F. xenicus*	38.5	35.0	2,449	5,733; n = 1,373,426	36.42	2,699	ERR3385273	ERR3385269
*F. catenatus*	40.3	36.6	1,699	3,439; n = 2,687,295	34.28	2,021	ERR3385274	ERR3385270
*F. nottii*	33.4	30.4	6,480	12,995; n = 700,534	31.06	7,548	ERR3385275	ERR3385271
*F. olivaceus*	50.1	45.5	4,595	11,670; n = 987,921	45.97	5,365	ERR3385276	ERR3385272

Coverage assumes that the genome size of each species is 1.1 Gb, as estimated for *F. heteroclitus* [40]. Untrimmed reads were deposited in the ENA under study PRJEB29136. Reads N50 represent the N50 length of all ONT reads before filtering and assembly, followed by the number (n) of reads constituting 50% of the length of all ONT reads. Data used for subsequent genome assemblies were filtered with a requirement for having a mean Phred quality score >Q5. The remaining bases called and mean read length that are >Q5 are listed.

**Table 2:** Illumina data collected were all paired-end 150 reads

Species	Platform	Reads (M)	Coverage (×)	FASTQ accessions
*F. xenicus*	Illumina HiSeq	327.5	89.3	ERR3385278
				ERR3385279
*F. catenatus*	Illumina HiSeq	316.5	86.3	ERR3385280
				ERR3385281
*F. nottii*	Illumina HiSeq	197.0	53.7	ERR3385282
				ERR3385283
*F. olivaceus*	Illumina NovaSeq	601.9	164.0	ERR3385284
				ERR3385285

Coverage assumes 1.1 Gb genome size measured for *F. heteroclitus* [40].

**Figure 3:**
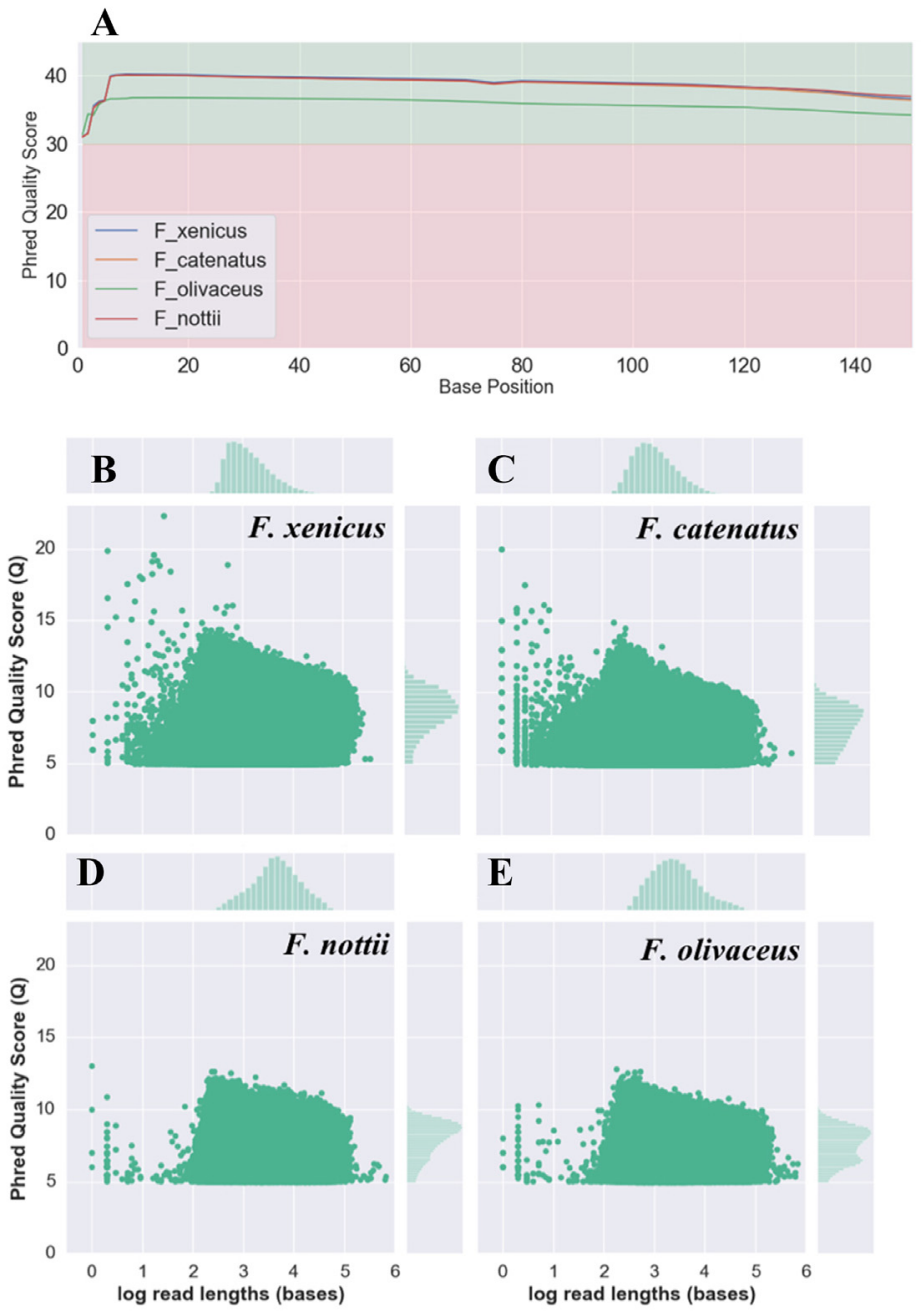
(A) Quality score profiles for representative R1 Illumina reads from *F. xenicus, F. catenatus, F. nottii*, and *F. olivaceus*. For Illumina data, phred quality scores were consistently above Q30 across all reads. Average read quality scores (Q score) vs. read lengths for ONT PromethION from (B) *F. xenicus*, (C) *F. catenatus*, (D) *F. nottii*, (E) *F. olivaceus*.

Mean quality scores for all Illumina data were consistently >Q30 (Fig. 3A). Residual Nextera adapters and bases with low quality scores were removed from Illumina reads using Trimmomatic PE (version 0.38) with conservative parameters, which included removing bases from each read with a quality score <Q2 and required a minimum read length of 25 bases each [45]. There did not appear to be a difference in the data quality (Fig. 3) when the hmw DNA was extracted from flash-frozen (*F. olivaceus* and *F. catenaus*) or fresh tissue (*F. xenicus* and *F. nottii*) (Fig. 2).

For *F. xenicus* and *F. catenatus*, ONT read qualities ranged from Q5 (minimum cut-off) to Q14, with read lengths generally ranging from 10 bp to 100 kb (Fig. 3B and C). For *F. nottii and F. olivaceus*, ONT read qualities ranged from Q5 (minimum cut-off) to Q13, with read lengths ranging from 100 bp to 100 kb (Fig. 3D and E).

### Draft assemblies

As a comparison with assemblies using long-read ONT data, Illumina data alone were assembled using ABySS (ABySS, RRID:SCR_010709) version 2.1.5. While the BUSCO scores were consistently >50% completeness [46], the number of contigs and contig N50 lengths of the Illumina-only assemblies were not acceptable for downstream use (Table 3).

**Table 3:** Statistics for Illumina-only assemblies using ABySS (version 2.1.5) for each species

Species	Bases in the Illumina-only assembly	N contigs	Mean length	Largest contig	N50	Illumina-only BUSCO C; CS/CD/F/M
*F. xenicus*	1,283,257,056	5,195,861	246.98	71,596	2,571; n = 107,350	57.1%; 56.4/0.7/33.3/9.6
*F. catenatus*	1,205,429,912	3,989,534	302.15	70,870	3,629; n = 80,839	53.8%; 52.8/1.0/36.0/10.2
*F. nottii*	1,167,835,004	3,875,693	301.32	92,540	3,740; n = 72810	62.7%; 61.7/1.0/27.4/9.9
*F. olivaceus*	1,252,948,998	4,509,089	277.87	70,765	3,670; n = 77136	65.7%; 64.0/1.7/25.1/9.2

The BUSCO Eukaryota database (303 genes) was used to evaluate the completeness of each assembly [46]. BUSCO numbers reported are percentage complete (C) followed by the percentages of complete single-copy (CS), complete duplicated (CD), fragmented (F), and missing (M) out of 303 genes.

The ONT-only assemblies using the fuzzy de Bruijn graph assembler, wtdbg2 (wtdbg2, RRID:SCR_017225) version 2.3 [47], had high contig N50 but low complete matches with the BUSCO Eukaryota database (Table 4). The assembler wtdbg2 took an average of 6.1 wall time hours per assembly and required 59 GB RAM. The polishing tool pilon required an average of 65.99 wall time hours and used 1.61 TB RAM. Following polishing with Illumina data using the pilon software tool version 1.23 [33], the BUSCO Eukaryota completeness scores increased to consistently >90% (Table 4). Compared with the BUSCO result for the existing assembly for *F. heteroclitus* (NCBI GCA_000826765.1 Fundulus_heteroclitus-3.0.2), which was 92.4% complete (CS: 89.8%, CD: 2.6%, F: 2.3%, M: 5.3%), the BUSCO results for these 4 species are quite complete. Assemblies were deposited in the Open Science Framework (OSF) repository [48] and zenodo record [49].

**Table 4:** ONT PromethION assemblies using the wtdbg2 version 2.3 assembler [47] followed by polishing with pilon version 1.23 [33]

Species	Contigs	Contig N50	Assembly size (bases)	Complete BUSCO C; CS/CD/F/M	
				After wtdbg2 ONT-only	After pilon polishing
*F. xenicus*	5,621	888,041; n = 325	1,075,031,690	10.2%; 10.2/0/11.6/78.2	90.5%; 87.5/3.0/3.0/6.5
*F. catenatus*	5,854	436,102; n = 780	1,163,592,740	11.2%; 28.4/0/24.4/47.2	90.4%; 88.4/2.0/2.6/7.0
*F. nottii*	2,242	2,701,963; n = 95	1,081,276,623	28.4%; 11.2/0/22.1/66.7	94.4%; 92.1/2.3/1.0/4.6
*F. olivaceus*	2,622	2,669,230; n = 105	1,198,526,423	23.4%; 23.4/0/25.7/50.9	92.1%; 89.8/2.3/1.3/6.6

Of interest is the dramatic improvement of the complete BUSCO metric after polishing with pilon. BUSCO numbers reported are percentage complete (C) followed by the percentages of complete single-copy (CS), complete duplicated (CD), fragmented (F), and missing (M) out of the 303 genes in the BUSCO Eukaryota database [46].

## Discussion

In this study, we collected 30–45× coverage of ONT data in combination with 50–160× coverage of Illumina PE150 sequencing data and generated draft genome assemblies for 4 species of *Fundulus* killifish. For the 4 assemblies presented here, the combination of ONT and Illumina data allowed us to generate highly contiguous assemblies with acceptable BUSCO results. The assemblies generated by ONT data alone were not acceptable for use because of the poor BUSCO results, likely due to the high rate of ONT sequence errors. Polishing the ONT assemblies with Illumina data did not improve contiguity of the assemblies but served to correct bases, fix misassemblies, and fill gaps, shown by the large boost in BUSCO scores relative to the ONT assemblies alone. However, even with improved BUSCO scores, assemblies may have high remaining indel rates due to problems inherent in mapping short Illumina reads to repetitive sequences [50].

The Phred base quality scores and the read lengths of the ONT data appeared to make a difference in the contig N50 metrics of the assemblies. Both *F. xenicus* and *F. catenatus* had shorter mean read lengths and reads N50 compared with *F. nottii* and *F. olivaceus*. The contig N50 metric for both *F. nottii* and *F. olivaceus* assemblies was larger (>2 Mb) compared with assemblies from *F. xenicus* and *F. catenatus* (<1 Mb). The assembly from *F. nottii*, which had the lowest data yield, had higher mean read lengths and higher reads N50 compared with the other species. *F. olivaceus*, which had the highest yield, also had a higher reads N50 and mean read length. Therefore, when generating ONT data for draft genome assemblies, the length and the quality of the reads may matter more than the overall yield of data. This is not easily controlled, except with the quality of the input hmw DNA sample, the quality of the ONT sequencing library and flow cell (Supplemental Fig. 1).

We observed lower yields from DNA isolated from our killifish samples compared with similar length DNA isolated from mammalian cultured cell lines. These lower yields are a result of a rapid decline in the active number of pores (Supplemental Fig. 1) possibly because of pore blockage. For the sample from *F. olivaceus*, we performed a nuclease flush and reloaded a second aliquot of the library that helped us improve the yield. Recent improvements in the unblock mechanisms in the MinKnow software along with nuclease flush can help to mitigate the blocking issue. The duty time plot (Supplemental Fig. 1) shows 60% pore occupancy at the beginning of the run, which then decreased to ∼18% in 17–18 hours. This was typical of the runs with all of the samples. Through our informal conversations with colleagues this appears to be a known problem in the nanopore community, at least for DNA from marine fish and birds. DNA isolated from these 4 killifish samples was fragile and easy to degrade as indicated by small fragments below 40 kb in the gel images. We suspect that this fragile DNA as well as pore blockage could be the cause of shorter read lengths and lower yields observed in our runs.

The Vertebrate Genome Project (VGP) lists standards for *de novo* genome assembly that include 4 types of data: Pacific Biosciences long reads, 10x Genomics linked Illumina reads, Hi-C chromatin mapping, and Bionano Genomics optical maps [51]. Each of these 4 types of data has associated costs of generation, including analysis and computational time. While chromatin capture and Hi-C methods produce high-quality chromosome-level assemblies [51–54], these data types can significantly increase the overall cost of the genome sequencing project. In this study, we report the pairing of just 2 data sets: short Illumina reads with long reads from the ONT PromethION platform, to generate a draft assembly at a lower cost. The qualities of the assemblies presented here are not as high compared to the standards recommended by the VGP, which requires the assembly to be haplotype phased with a minimum contig N50 of 1 Mb, scaffold N50 of 10 Mb, 90% of the genome assembled into chromosomes, and a sequence error frequency of ≥Q40 [51]. However, the assemblies presented here and for *F. heteroclitus* [40] are sufficient for many uses. For *F. olivaceus* and *F. nottii*, draft assemblies using wtdbg2 [47] and pilon polishing with Illumina data [33] had contig N50 >1 Mb, which meets the minimum requirements for assemblies in downstream synteny analyses [11].

New software tools and methods for base-calling, assembling, and analyzing noisy ONT long reads are being developed at a fast rate [55, 56]. Because of this fast pace of software tool development for ONT data, standard operating procedures are not available. While we intend to use the 4 assemblies presented here for comparative evolutionary analyses, the raw data are shared here with the intent that others may use them for tool development and as new workflow pipelines, algorithms, tools, and best practices emerge.

## Conclusions

Sequencing data from the ONT PromethION and Illumina platforms combined can contribute to assemblies of eukaryotic vertebrate genomes (>1 Gb). These sequencing data from wild-caught individuals of *Fundulus* killifish species are available for use with tool development and workflow pipelines. Ongoing work from our group is comparing genomic content between these *Fundulus* species to address questions about evolutionary mechanisms of divergence between marine and freshwater niches.

## Data Reuse Potential

We encourage use and reuse of these data. This collection of whole-genome sequencing data from the PromethION and Illumina platforms originates from wild-caught individuals of closely related *Fundulus* killifish species, obtained for the purpose of comparative evolutionary genomics analyses. These data, which add to the growing set of public data available from the ONT PromethION sequencing platform [25, 57], can be used for developing base-calling and assembly algorithms.

## Availability of Supporting Data and Materials

Raw data are available in the ENA under study PRJEB29136. Draft assembly data products and quality assessment reports are available in the OSF repository [48] and zenodo [49]. Scripts used for this analysis workflow are available at ONT_Illumina_genome_assembly [58]. All supporting data and materials are available in the *GigaScience* GigaDB database [59].

## Additional Files

**Supplemental Table 1**. Flow cell and base-caller version summary for all the PromethION sequencing runs.

**Supplemental Figure 1**. Duty time plot of the *F. nottii* sequencing run on PromethION indicating rapid decline in the number of active pores. This plot was typical of all samples. The pore occupancy started at 60% then decreased to <20% in 17 hours.

giaa067_GIGA-D-19-00351_Original_Submission

giaa067_GIGA-D-19-00351_Revision_1

giaa067_Response_to_Reviewer_Comments_Original_Submission

giaa067_Reviewer_1_Report_Original_Submission Chao Bian -- 10/30/2019 Reviewed

giaa067_Reviewer_1_Report_Revision_1 Chao Bian -- 4/21/2020 Reviewed

giaa067_Reviewer_2_Report_Original_Submission Jason R. Miller, MS -- 10/30/2019 Reviewed

giaa067_Reviewer_3_Report_Original_Submission Christiaan Henkel -- 10/31/2019 Reviewed

giaa067_Supplemental_File

## Abbreviations

bp: base pairs; BUSCO: Benchmarking Universal Single-Copy Orthologs; EDTA: ethylenediaminetetraacetic acid; ENA: European Nucleotide Archive; Gb: gigabase pairs; hmw: high molecular weight; IACUC: Institutional Animal Care and Use Committee; kb: kilobase pairs; Mb: megabase pairs; NCBI: National Center for Biotechnology Information; ONT: Oxford Nanopore Technologies; OSF: Open Science Framework; PE: paired end; RAM: random access memory; SDS: sodium dodecyl sulfate; VGP: Vertebrate Genome Project.

## Ethical Approval

UC Davis IACUC protocol No. 17221.

## Competing Interests

The authors declare that they have no competing interests.

## Funding

Gordon and Betty Moore Foundation to C.T.B. under award No. GBMF4551. IU-TACC Jetstream and PSC Bridges XSEDE allocations TG-BIO160028 and TG-MCB190015 to L.K.J.

## Authors' Contributions

Sample extractions and library preparations were done by L.K.J., R.S., J.A.G., and J.L.R. Project advising was by C.T.B. and A.W. Manuscript writing and editing were by L.K.J., R.S., J.A.G., J.L.R., L.F., C.T.B., and A.W.
