# Supplementary material for: Draft genome assemblies using sequencing reads from Oxford Nanopore Technology and Illumina platforms for four species of North American Fundulus killifish
Source: Gigascience. 2020 Jun 18;9(6):giaa067. doi: 10.1093/gigascience/giaa067 (PMC7301629; doi:10.1093/gigascience/giaa067)
Supplement: giaa067_GIGA-D-19-00351_Revision_1 [file giaa067_giga-d-19-00351_revision_1.pdf]

GigaScience

Draft genome assemblies using sequencing reads from Oxford Nanopore Technology and Illumina platforms for four species of North American Fundulus killifish

--Manuscript Draft--

**Manuscript Number:**	GIGA-D-19-00351R1
**Full Title:**	Draft genome assemblies using sequencing reads from Oxford Nanopore Technology and Illumina platforms for four species of North American Fundulus killifish
**Article Type:**	Data Note
**Funding Information:**	Gordon and Betty Moore Foundation (GBMF4551) — Dr C. Titus Brown
**Abstract:**	Background<br>Whole genome sequencing data from wild-caught individuals of closely-related North American killifish species (Fundulus xenicus , Fundulus catenatus , Fundulus nottii , and Fundulus olivaceus) were obtained using long-read Oxford Nanopore Technology (ONT) PromethION and short-read Illumina platforms.<br>Findings<br>Draft de novo reference genome assemblies were generated using a combination of long and short sequencing reads. For each species, the PromethION platform was used to generate 30-45x sequence coverage, and the Illumina platform was used to generate 50-160x sequence coverage. Illumina-only assemblies were fragmented with high numbers of contigs while ONT-only assemblies were error prone with low BUSCO scores. The highest N50 values, ranging from 0.4 Mb to 2.7 Mb, were from assemblies generated using a combination of short and long read data. BUSCO scores were consistently above 90% complete using the Eukaryota database.<br>Conclusions<br>Good quality genomes can be obtained from a combination of using short read Illumina data to polish assemblies generated with long read ONT data. Draft assemblies and raw sequencing data are available for public use. We encourage use and re-use of these data for assembly benchmarking and other analyses.
**Corresponding Author:**	Andrew Whitehead<br>University of California Davis<br>Davis, CA UNITED STATES
**Corresponding Author Secondary Information:**	
**Corresponding Author's Institution:**	University of California Davis
**Corresponding Author's Secondary Institution:**	
**First Author:**	Lisa Kristine Johnson
**First Author Secondary Information:**	
**Order of Authors:**	Lisa Kristine Johnson
	Ruta Sahasrabudhe
	James Anthony Gill
	Jennifer L. Roach
	Lutz Froenicke
	C. Titus Brown
	Andrew Whitehead
**Order of Authors Secondary Information:**	
**Response to Reviewers:**	We thank the reviewers for their time and for providing these comments, which have improved this Data Note paper.

Powered by Editorial Manager® and ProduXion Manager® from Aries Systems Corporation

Reviewer #1:

>The authors from this paper "Draft genome assemblies using sequencing reads from Oxford Nanopore Technology and Illumina platforms for four species of North American killifish from the Fundulus genus" has reported four species of famous Fundulus killifish assembled by the combination of ONT data and Illumina data. The quality of these four assemblies was pretty well. Their BUSCO results also showed high completeness. The assembling pipeline was described in detail.
>However, the language of this article should be gone through carefully. Too much errors were in the manuscript. As well as, only assembly data were really limited.

Thank you for your comments on our 4 killifish genome assemblies using ONT and Illumina sequencing data. We have fixed the errors you have found; thank you for bringing them to our attention. We have carefully revised the language of the paper according to your recommendations.

>For the abstract: the format should be revised for gigascience publication.

Done. As specified by the GigaScience Data Note instructions for text formatting, we have separated the abstract into three separate sections: Background, Findings, and Conclusions

>Key words: "genome assembly" should be genome assemblies.

We have changed this keyword to “genome assemblies”

>Lines 32-35, this sentence is too heavy, please split it to be clearer.

We have changed this sentence from:

Single-molecule long read nucleic acid sequencing technology from Oxford Nanopore Technologies (ONT), which has been commercially available since 2014 [7], has been shown to improve the contiguity of reference assemblies [8] and reveal “dark regions” that were previously camouflaging genes [9].

to

Single-molecule long read nucleic acid sequencing technology from Oxford Nanopore Technologies (ONT) has been commercially available since 2014 [7]. This technology has been shown to improve the contiguity of reference assemblies [8] and reveal “dark regions” that were previously camouflaging genes [9].

>Line 56, remove a "genome" from "genome genome assembly".

We have removed this redundant word.

>The decimal place of number should be uniform through whole article, 91.2 Gb in line 50, 110.96 Gb in line 52 and 70.6 Gb in 63 should be changed.

We changed the number 110.96 to 111.0 Gb.

>Line 72, "after polishing" should be changed to "after being polished".

We have made this correction.

>Line 77, how about "repeated genomic divergence". It means multiple genomic divergence?

We have changed this sentence from:

Fundulus is a comparative evolutionary model system 76 for studying repeated genomic divergence between marine and freshwater species. Fundulus 77 killifish have a cosmopolitan geographic distribution across North America.

Powered by Editorial Manager® and ProduXion Manager® from Aries Systems Corporation

to

Fundulus is a comparative model system for studying genomic divergence that has repeated independently multiple times throughout evolutionary history between marine and freshwater species.

>Line 91, what's the real meaning of "the functional basis of, and evolution of,"?

We have removed this sentence since it is not relevant to the manuscript. Readers who would like more information about this statement are invited to see the citations listed.

The manuscript now reads:

The Atlantic killifish, Fundulus heteroclitus has been a well-described model organism for investigating physiological resilience to temperature, salinity, hypoxia, and environmental pollution [34,37–39]. There is a reference genome available for F. heteroclitus [40]. However, we do not currently have any reference genomes from other Fundulus killifish, particularly from those occupying freshwater habitats.

>For the Figure 1, the quality of this figure is too poor, please revise.

We apologize for the low quality resolution in Figure 1. Thank you for bringing this to our attention. High resolution images will be provided to the publisher.

>Line 128, for the "both the ultra-long read sequencing protocol from [42]", "from" what?

We have changed this sentence from:

For 127 F. catenatus and F. olivaceus, both the ultra-long read sequencing protocol from [42] (which 128 included Tissue lysis buffer with Tris, NaCl, EDTA, SDS and Proteinase K followed by 129 phenol:chloroform extraction), as well as the Qiagen "DNA purification from tissue using the 130 Gentra puregene Tissue Kit" (p. 39) were used, and were found to be similar to the Agilent kit.

to

For F. catenatus and F. olivaceus, two extraction methods were tested: 1) Tris, NaCl, EDTA, SDS and Proteinase K followed by phenol:chloroform extraction [42] and 2) the Qiagen "DNA purification from tissue using the Gentra puregene Tissue Kit" (p. 39).

>Line 142, "tissue" should tissues.

We have made this correction.

>Line 150, "reads N50" should be changed to "read N50".

We feel that we need to keep this wording to make the distinction between ONT reads N50 as opposed to genome assembly N50.

>Only the genome assemblies are indeed limited for further using, like comparative genomic analysis. This will seriously impede the power of this important article and these famous species. I strongly suggest the authors to perform the genome annotation of these four killifish, and re-submit this article.

Thank you for this suggestion. We agree that annotations will be a valuable resource for these genomes and for comparative analyses. However, generating accurate and reliable annotations for new genome assemblies requires considerable additional work. Given the usefulness of these new genome assemblies and raw PromethION sequencing data to the broader research community, we chose to contribute a Data Note, which makes these data immediately accessible. An annotation effort is ongoing by our research group and others are welcome to also pursue annotation.

Reviewer #2:

Powered by Editorial Manager® and ProduXion Manager® from Aries Systems Corporation

We thank Reviewer #2 for their comments and observation that the data are accessible online.

>As is appropriate for a Data Note, the manuscript lacks the analysis that is expected of a genomes paper. It does not present coverage analysis, which could indicate areas of mis-assembly. It does not include the gene annotation of any assembly. It does not include any alignments between the four new assemblies or the previously published one. It does not speculate on possible causes of the six-fold variation in contig N50.

Yes, this is accurate. The manuscript does lack the analysis expected of a genome paper, and does not present mis-assembly coverage analysis, annotations, comparative genomic analyses, or assembly quality analysis beyond BUSCO scores.

>1. The tables and figures could use some adjustment to increase their value to readers.
>The caption to Table 1 should explain or reference the N50 vs n statistics in column 5, and the Q>5 threshold in columns 6 and 7. The caption to Figure 3 should label the five colored wiggle plots that appear in 3A. Table 2 should use significant digits consistently. Table 3 should list the species in the same order as the other tables for ease of comparison.

Thank you for bringing this to our attention. We made the following adjustments to the tables and figures:

•We added this to the caption to Table 1:

Reads N50 represent the N50 length of all ONT reads before filtering and assembly; followed by the number (n) of reads constituting 50% of the length of all ONT reads. Data used for subsequent genome assemblies were filtered with a requirement for having an average Phred quality score >Q5). The remaining bases called and average read length that are >Q5 are listed.

•The Q vs. cycle plots in Figure 3A were adjusted to only include R1, for ease of labeling (reducing the number of lines from 14 down to 4 and a figure legend was added to indicate which lines correspond to which species. For full details of this plot, please see this notebook:
https://github.com/dib-lab/ONT_Illumina_genome_assembly/blob/master/qc/fastqc/plots_all_fadapa.ipynb

•We fixed the significant digits in Table 2 and changed order of species in all tables so that they are consistent.

>2. The discussion of cost should be more disciplined. This paper refers to "the low cost" of its assemblies in contrast to "high cost of generation" with other technologies. However, no cost data is presented or referenced. Prices are difficult to pin down but there must be some way to provide a helpful comparison backed by facts.

We agree that sequencing prices are difficult to pin down, e.g. what we paid might be different than another research group with access to different facilities. We edited the wording to generally discuss costs of data types, rather than specific costs. This paragraph now reads:

The Vertebrate Genome Project (VGP 2018) lists standards for de novo genome assembly that include four types of data: PacBio long reads, 10x linked Illumina reads, Hi-C chromatin mapping and Bionano Genomics optical maps. Each of these four types of data has associated costs of generation, including analysis and computational time. While chromatin capture and Hi-C methods produce high quality chromosome-level assemblies [48–51], these data types can significantly increase the overall cost of the genome sequencing project. Here, we report the pairing of high-accuracy short Illumina reads with error-prone long reads from the ONT PromethION platform (two out of the four VGP required sequencing datasets) to generate a draft assembly at a minimum cost. The qualities of these assemblies are not as high as compared to the standards recommended by VGP (2018) with the 3.4.2.QV40 phased metric, which

Powered by Editorial Manager® and ProduXion Manager® from Aries Systems Corporation

requires the assembly to be haplotype phased with a minimum contig N50 of 1 million bp (1Mb), scaffold N50 of 10Mb, 90% of the genome assembled into chromosomes and a base quality error of Q40, (VGP 2019). However, for many research purposes these assemblies are sufficient, considering that we have a high-quality reference genome assembly for another species within the genus [40]. For F. olivaceus and F. nottii, draft assemblies using wtdbg2 [47] and pilon polishing with Illumina data [33] had contig N50 >1 Mb, which meets the minimum requirements for assemblies in downstream synteny analyses [11].

>3. The title names two companies. Could it instead refer to polished nanopore assemblies?

We think that a title that specifically names the sequencing technologies will help readers to find the paper and accordingly would like to keep the current title.

We have added the word “polish” to the keywords and in the abstract, to highlight the importance of the polishing analysis on improving the quality of the genome assemblies.

>4. The discussion section should be revised to remove imprecise wording.
>* Quality standards of the VGP could use better explanation and citation.

We inserted a better citation for the Vertebrate Genome Project (2018):

Vertebrate Genome Project. A reference standard for genome biology. Nat Biotechnol. 2018;36:1121. Available from: http://dx.doi.org/10.1038/nbt.4318

>* The phrase "these assemblies" is ambiguous in places.

We changed the wording to: “For the four assemblies presented here”

>* Pilon "served to correct errors"; which kind?

We changed the wording to "served to correct bases, fix mis-assemblies and fill gaps", to be more specific about the corrections made with the Pilon program.

>* The "qualities of the ONT data appeared to make a difference"; which qualities?

We clarified the wording in this paragraph. We changed from:

The qualities of the ONT data appeared to make a difference in the contig N50 metrics of the assemblies. F. nottii and F. olivaceus both had contig N50 >2Mb, while assemblies from F. xenicus and F. catenatus had contig N50 <1Mb. F. xenicus and F. catenatus had shorter average read lengths and reads N50, on average, compared to F. nottii and F. olivaceus. F. nottii, which had the lowest yield, had higher average read lengths and reads N50 compared to the other species. F. olivaceus, which had the highest yield, also had a high reads N50 and average read length. Therefore, when generating ONT data for draft genome assemblies, it might matter more to have a lower yield of longer reads than a higher yield of shorter reads.

to:

The Phred base quality scores and the read lengths of the ONT data appeared to make a difference in the contig N50 metrics of the assemblies. Both F. xenicus and F. catenatus had shorter average read lengths and reads N50 compared to F. nottii and F. olivaceus. The contig N50 metric for both F. nottii and F. olivaceus was larger (>2 Mb) compared to F. xenicus and F. catenatus (<1 Mb). F. nottii, which had the lowest data yield, had higher average read lengths and higher reads N50 compared to the other species. F. olivaceus, which had the highest yield, also had a high reads N50 and average read length. Therefore, when generating ONT data for draft genome assemblies, the size and the quality of the reads matter more than the overall yield of data. This can be input quality of the DNA sample or the quality of the flow cell.

>* "It might matter more to have lower yield of longer reads"; matter how? Anyway, is

that a setting on the sequencer?

Please see the above change to this paragraph. We clarified by mentioning that the combination of the quality of the hmw DNA sample and the ONT library seem to make more of a difference for the read length and quality than the overall yield of data.

>* "Our samples were consistently not using pores as efficiently" seems to be a colloquialism.

We changed the wording to:

We observed lower yields from DNA isolated from our killifish samples compared to similar length DNA isolated from mammalian cultured cell lines. These lower yields are a result of a rapid decline in the active number of pores (Supplemental Figure 1) possibly because of pore blockage. For the sample from F. olivaceus, we performed a nuclease flush and re-loaded a second aliquot of the library that helped us improve the yield. Recent improvements in the unblock mechanisms in the MinKnow software along with nuclease flush can help to mitigate the blocking issue. The duty time plot (Supplemental Figure 1) shows 60% pore occupancy at the beginning of the run, which then dropped down to approximately 18% in 17-18 hrs. This was typical of the runs with all of the samples. Through our informal conversations with colleagues this appears to be a known problem in the nanopore community, at least for DNA from marine fish and birds.

>* A "brittle property" of the DNA is probably speculation.

DNA isolated from these four killifish samples was fragile and easy to degrade as indicated by small fragments below 40 kb in the gel images. This fragile DNA as well as the known ability of fish DNA to block pores could be the cause of shorter read lengths and lower yields observed in our runs. We changed our wording to "DNA isolated from these four killifish samples was fragile and easy to degrade as indicated by small fragments below 40 kb in the gel images. We suspect that this fragile DNA as well as pore blockage could be the cause of shorter read lengths and lower yields observed in our runs."

>* One paper's recommendation for synteny analysis may not warrant being called THE minimum requirements.

We clarified the wording to specifically mention synteny analysis and not overemphasize meeting all minimum requirements.

Reviewer #3:

>This is a useful study describing the sequencing and initial assembly of four Fundulus species. Although there is a lot of interesting genome biology to be discovered, this paper focuses on the technicalities of data collection only.

Thank you, we are glad you find this study useful. Yes, it is true that we have focused on the technical aspects of generating the data and the assemblies.

>To be useful for the intended benchmarking purposes, I would like to ask the authors to supply some additional details on several stages of the sequencing and analyses:
>P3, Methods and Results: How did you identify the species?

Fish samples were identified in the field by experts. We clarified by adding the following wording:

Live field-caught individuals of each fish species were identified by field experts, shipped to UC Davis and kept at their native salinities in an animal holding facility maintained according to University of California IACUC standards.

>P5, ONT sequencing: I assume you used R9.4(.1?) flowcells. Please list the exact types, as these have changed several times already and are about to change again. Also, which version of which ONT basecaller did you use?

Powered by Editorial Manager® and ProduXion Manager® from Aries Systems Corporation

The chart with flow cell and basecaller version summary for all the runs was added as Supplemental Table 1.

>P6, Table 1: Coverage is calculated using a reference species genome, but it should be straightforward to estimate it for each species based on Illumina k-mer profiles. The assembly sizes (Tables 3,4) vary by ~10%, it would be nice to know whether this reflects actual differences in genome size between the species. In addition, this will yield an estimate of heterozygosity, which is relevant for the interpretation of the Illumina assemblies (high heterozygosity may result in harder to assemble genomes when using short reads).

Thank you for the suggestion. We agree that an assembly-independent genome size estimation would be valuable and it would be nice to know whether the genome size differences in Tables 3,4 are due to actual differences between species or because of assembly errors. This is actually a non-trivial analysis requiring the use of additional software tools and computing resources. Given the usefulness of these new genome assemblies and the raw PromethION sequencing data to the broader research community, we chose to contribute a Data Note, which makes these data immediately accessible. Future genome size estimation could be part of future analysis using these genomes.

>P8/9, BUSCO scores: It would be very informative if you included a positive control here, by running BUSCO on the F. heteroclitus reference genome. (PS I am somewhat surprised the fragmented Illumina assemblies yield relatively decent completeness scores. Could this be a result of the specific reference database (eukaryotes) used? Perhaps strongly conserved genes are often found in regions of the genome that are relatively easy to assemble?)

Thank you for this suggestion. We performed the positive control BUSCO run and added the following:

Comparing to the BUSCO results for the existing assembly for sister F. heteroclitus (NCBI GCA_000826765.1 Fundulus_heteroclitus-3.0.2), which was 92.4% complete (CS:89.8%,CD:2.6%,F:2.3%,M:5.3%), the BUSCO results for these four species are quite complete.

>P9, l221: How did you run Pilon? Some recommend running it multiple times? Polishing will have changed the genome lengths/N50s listed in Table 4.

We ran several iterations of the pilon program to polish assemblies, as was recommended by the methods used in Miller et al. (2018): https://www.ncbi.nlm.nih.gov/pubmed/30087105 We found that these extra iterations of pilon did not change the quality of the assemblies so we have reported only one iteration with pilon. In general, the lengths of the contigs and the N50 scores did not change with pilon because it is correcting individual bases and not changing the continuity of the contigs.

>P10, Discussion: There is some variation in sample storage conditions, DNA isolation, library prep methods, etc. As far as I can see, there are no clear correlations with data and assembly quality, but perhaps you could summarize this here?

The following was added to summarize:

There did not appear to be a difference in the data quality (Figure 3) when the hmwDNA was extracted from fresh frozen (F. olivaceus and F. catenaus) or fresh tissue (F. xenicus and F. nottii) (Figure 2).

Some minor thoughts & comments:

>P2, l49: Although PromethION has higher throughput than MinION, this section also compares ONT data from different timepoints. Changes in chemistry and protocols are probably of greater influence on the increases in throughput than the platform. Today, MinION throughput is (under good conditions) higher than the 6.5 Gbp mentioned.

Powered by Editorial Manager® and ProduXion Manager® from Aries Systems Corporation

	Yes, the improvements with pore chemistry and protocols are increasing the yield with MinION. However, the pore chemistry is inherently different in the PromethION because of the higher density of pores on the flow cells. We have added this to the end of the paragraph to clarify:<br><br>While changes in pore chemistry and protocols are improving the yields from the ONT MinION, the yield from the ONT PromethION platform remains higher because of the higher density of nanopore channels.<br><br>>P2, l67, 'Recently…': This approach using Illumina polishing is not of a more recent date than the scaffolding method. Fish genomes produced using either method were published in 2017 already.<br><br>We removed the word "recently" from this sentence.<br><br>>P3, l89: This sentence introduces the Atlantic killifish twice.<br><br>This redundancy was removed.<br><br>P9, l220: I assume the 6.1 hours for wtdbg2 are not CPU hours (as listed for Pilon, next line)? I have used it to assemble a similar genome, which took ~350 CPU hours.<br><br>Yes, we clarified that the hours reported are wall time hours, not CPU hours.<br><br>>P10, Discussion, third paragraph: DNA from fatty fish is notoriously difficult to sequence, with low yields due to rapid pore deaths. I think the cause remains unknown (and the phenomenon unpublished?). Although the fish sequenced here are not that rich in oils, did you observe the same phenomenon (i.e. when you mention 'not using pores as efficiently')?<br><br>Yes, the cause of low yields due to rapid pore deaths remains unknown. We have searched the literature and have not seen anything related to this, so felt that it was important to document here what we observed.
**Additional Information:**	
**Question**	**Response**
Are you submitting this manuscript to a special series or article collection?	No
**Experimental design and statistics**<br><br>Full details of the experimental design and statistical methods used should be given in the Methods section, as detailed in our Minimum Standards Reporting Checklist. Information essential to interpreting the data presented should be made available in the figure legends.<br><br>Have you included all the information requested in your manuscript?	Yes
**Resources**	Yes

Powered by Editorial Manager® and ProduXion Manager® from Aries Systems Corporation

A description of all resources used, including antibodies, cell lines, animals and software tools, with enough information to allow them to be uniquely identified, should be included in the Methods section. Authors are strongly encouraged to cite Research Resource Identifiers (RRIDs) for antibodies, model organisms and tools, where possible.<br><br>Have you included the information requested as detailed in our Minimum Standards Reporting Checklist?	
**Availability of data and materials**<br><br>All datasets and code on which the conclusions of the paper rely must be either included in your submission or deposited in publicly available repositories (where available and ethically appropriate), referencing such data using a unique identifier in the references and in the "Availability of Data and Materials" section of your manuscript.<br><br>Have you have met the above requirement as detailed in our Minimum Standards Reporting Checklist?	Yes

Powered by Editorial Manager® and ProduXion Manager® from Aries Systems Corporation

Manuscript Click here to access/download;Manuscript;351-final.docx

Draft genome assemblies using sequencing reads from Oxford Nanopore Technology and Illumina platforms for four species of North American *Fundulus* killifish

Lisa K. Johnson [1,2], Ruta Sahasrabudhe [3], James Anthony Gill [1], Jennifer L. Roach [1], Lutz Froenicke [3], C. Titus Brown [2], Andrew Whitehead* [1]

[1] Department of Environmental Toxicology, University of California, Davis
[2] Department of Population Health & Reproduction, School of Veterinary Medicine, University of California, Davis
[3] DNA Technologies Core, Genome Center, University of California, Davis
*corresponding author: awhitehead@ucdavis.edu

ORCIDs:
Lisa K. Johnson, 0000-0002-3600-7218;
Ruta Sahasrabudhe, 0000-0002-3285-6845;
C. Titus Brown, 0000-0001-6001-2677.

Abstract

Background
Whole genome sequencing data from wild-caught individuals of closely-related North American killifish species (*Fundulus xenicus*, *Fundulus catenatus*, *Fundulus nottii*, and *Fundulus olivaceus*) were obtained using long-read Oxford Nanopore Technology (ONT) PromethION and short-read Illumina platforms.

Findings
Draft *de novo* reference genome assemblies were generated using a combination of long and short sequencing reads. For each species, the PromethION platform was used to generate 30-45x sequence coverage, and the Illumina platform was used to generate 50-160x sequence coverage. Illumina-only assemblies were fragmented with high numbers of contigs while ONT-only assemblies were error prone with low BUSCO scores. The highest N50 values, ranging from 0.4 Mb to 2.7 Mb, were from assemblies generated using a combination of short and long read data. BUSCO scores were consistently above 90% complete using the Eukaryota database.

Conclusions
Good quality genomes can be obtained from a combination of using short read Illumina data to polish assemblies generated with long read ONT data. Draft assemblies and raw sequencing data are available for public use. We encourage use and re-use of these data for assembly benchmarking and other analyses.

Keywords: long reads; Oxford Nanopore; killifish; genomes; genome assemblies; polish

Background

Sequencing and assembling large eukaryotic genomes is challenging [1–3]. Accuracy of downstream analyses, such as selection scans, synteny analysis, and measuring gene expression, require high-quality reference genome assemblies [4]. Fortunately, as costs of generating whole genome sequence data drop, it is becoming easier for individual labs rather than large consortiums to generate assemblies for organisms without reference genomes [3,5,6]. Single-molecule long read nucleic acid sequencing technology from Oxford Nanopore Technologies (ONT) has been commercially available since 2014 [7]. This technology has been shown to improve the contiguity of reference assemblies [8] and reveal "dark regions" that were previously camouflaging genes [9]. The lengths of the sequencing reads generated using this technology are limited only by the size of the fragments in the extracted DNA sample [10]. The promise of more complete reference assemblies is especially important for the accuracy of comparative evolutionary genomics studies, as assembly fragments lead to errors in downstream synteny analyses [11], as well as SNP calling and identification of transcript features (splice junctions and exons) for quantification.

Despite high error rates of ONT reads ~5% [12] relative to Illumina short reads ~0.3% [13] and the relatively recent availability of ONT data, there has been recent expansion of genome projects using this sequencing technology. Small genomes from bacteria and viruses appear to be ideal for sequencing on the ONT MinION platform [12]. The portable nature of the technology makes it appealing as a resource for teaching [14,15], working in remote locations [16–18] and for investigating viral outbreak public health emergencies [19–21]. However, despite the demonstrated ability to achieve yields >6.5 Gb per flow cell [22], the MinION platform can be prohibitively expensive for sequencing larger eukaryotic genomes. For example, 39 flow cells yielded 91.2 Gb of sequence data (~30x coverage) of the human genome [23].

Sequencing of the wild tomato species *Solanum pennellii* across thirty-one flow cells yielded 111.0 Gb (~100x coverage) with some flow cells yielding >5 Gb [24]. By contrast, following the 2018 beta release of the ONT PromethION platform, which has a higher density of nanopore channels, five flow cells were used to yield >250.0 Gb (~80x coverage) of the human genome [25]. PromethION data combined with Hi-C long-range mapping data from human samples produced a genome assembly with a scaffold N50 of 56.4 Mb [26]. While changes in pore chemistry and protocols are improving the yields from the ONT MinION, the yield from the ONT PromethION platform is larger because of the higher density of nanopore channels.

The combination of long read sequencing data from ONT MinION and short read sequencing data from Illumina has been used to improve the quality of reference genomes [27–30]. In one approach, short read assembly scaffolds have been improved with the addition of long reads. The Murray cod genome (640-669 Mb in size) was improved by combining low coverage (804 Mb) of long reads ONT data from just one MinION flow cell with 70.6 Gb of Illumina data from both HiSeq and MiSeq; the assembly scaffold N50 increased from 33,442 bp (Illumina only) to 52,687 bp with ONT and Illumina combined [31]. The clownfish genome (791 to 794 Mb in size) was improved by including 8.95 Gb of ONT MinION reads; the scaffold N50 increased from 21,802 bp (Illumina only) to 401,715 bp with ONT and Illumina combined [27]. Consensus building with racon [32] and/or pilon [33] tools use Illumina data to “polish” contigs from ONT-only assemblies. Polishing corrects single nucleotide base differences, fills gaps, and identifies local mis-assemblies [33]. This approach has been shown to improve the BUSCO score from <1% with the ONT assembly alone to >95% complete after polishing with Illumina reads, with significant reduction of indels and homozygous and heterozygous SNPs [28].

In this study, we explored whether the ONT PromethION sequencing technology could be appropriate for generating draft reference genomes for four species of North American killifish belonging to the *Fundulus* genus. *Fundulus* is a comparative model system for studying evolutionary divergence between marine and freshwater environments. *Fundulus* killifish are broadly distributed across North America. These small cyprinodontiform fish have evolved to occupy a wide range of osmotic niches, including marine, estuarine, and freshwater [34]. Estuarine and coastal *Fundulus* are euryhaline, insofar as they can adjust their physiologies to tolerate a very wide range of salinities. In contrast, freshwater species are stenohaline: they tolerate a much narrower range of salinities [34,35]. Freshwater clades are derived from marine clades, and radiation into freshwater has occurred multiple times independently within the genus. This makes *Fundulus* unusual, because most large clades of fishes are either exclusively marine or exclusively freshwater. Therefore, species of closely-related killifish in the *Fundulus* genus serve as a unique comparative model system for understanding the genomic mechanisms that contribute to evolutionary divergence and convergence of osmoregulatory processes, which is important for understanding how species will cope with changing salinity regimes expected with climate change [36]. The Atlantic killifish, *Fundulus heteroclitus* has been a well-described model organism for investigating physiological resilience to temperature, salinity, hypoxia, and environmental pollution [34,37–39]. There is a reference genome available for *F. heteroclitus* [40]. However, no reference genomes exist from other *Fundulus* killifish, particularly from those occupying freshwater habitats.

Here, we report the collection of whole genome sequencing data using both ONT PromethION and Illumina platforms from four killifish species without previously-existing sequencing data (Figure 1): *Fundulus xenicus* (NCBI:txid722643, Fishbase ID: 3166; formerly

Adinia xenica) [41], *Fundulus catenatus* (NCBI:txid34776, Fishbase ID: 3186), *Fundulus nottii* (NCBI:txid54270, Fishbase ID: 3198), and *Fundulus olivaceus* (NCBI:txid34782, Fishbase ID: 3199). *F. xenicus* is euryhaline and occupies coastal and estuarine habitats, while the other species (*F. catenatus*, *F. nottii*, *F. olivaceus*) are stenohaline and occupy freshwater habitats.

Methods and Results

Live field-caught individuals of each fish species were identified by field experts, shipped to UC Davis and kept at their native salinities in an animal holding facility maintained according to University of California IACUC standards. *F. catenatus* and *F. olivaceus* were collected from the Gasconade River, MO (latitude/longitude coordinates 37.879/-91.795 and 37.19/-92.56, respectively), *F. nottii* was collected from Walls Creek, MS (31.154433/-89.245381), and *F. xenicus* was collected from Graveline Bayou, MS (30.368756/-88.719329). High molecular weight (hmw) DNA was extracted from fresh tissue for *F. nottii* and *F. xenicus*, and from frozen tissue for *F. catenatus* and *F. olivaceus*. For *F. catenatus* and *F. olivaceus*, tissues were dissected and frozen in liquid nitrogen then stored immediately at -80 °C until samples were prepared for hmw DNA extraction. With the exception of *F. olivaceus*, each assembly consisted of sequencing one tissue sample from one individual. For *F. olivaceus*, Illumina data were collected from DNA extracted from one individual while the ONT PromethION data were collected from another individual (frozen tissue).

DNA extractions

Whole fish heads were used for hmw DNA extractions. Agilent’s Genomic DNA Isolation kit (Catalog #200600) was used to extract DNA from fresh tissues from *F. xenicus* and

F. nottii. For *F. catenatus* and *F. olivaceus*, two extraction methods were tested: 1) Tris, NaCl, EDTA, SDS and Proteinase K followed by phenol:chloroform extraction [42] and 2) Qiagen's Gentra Puregene Tissue Kit (Catalog #158667). These were both found to be similar to the Agilent kit. Precipitated DNA was difficult to re-dissolve; therefore, additional phenol:chloroform cleanup steps were added after extractions. We found that adding urea to the lysis buffer helped to precipitate the DNA pellet, making it less fragile and go into solution easier [43]. Prior to library preparation, hmw DNA from *F. nottii* and *F. olivaceus* (PromethION) was sheared to 50 kb in an effort to improve the ligation enzyme efficiency, resulting in fragments in the 50-70 kb range. Field inversion gels were used to visualize hmw DNA (Figure 2).

ONT sequencing

Libraries for ONT PromethION sequencing were prepared using the ligation sequencing kit (SQK-LSK109) following the manufacturer's instructions. ONT PromethION sequencing data were collected from all four species on an alpha-beta instrument through the early release program at the University of California, Davis DNA Technologies Core facility (Davis, CA USA). One species was sequenced per R9.4 flow cell (PRO001 and PRO002). Base-calling was done onboard the PromethION instrument using MinKnow versions 2.0-2.2 (Oxford Nanopore Technologies, UK). Flow cell and basecaller versions can be found in Supplemental Table 1. For the *F. xenicus* run, lambda phage (DNA CS) was spiked-in as a positive control.

Illumina Sequencing

With the exception of *F. olivaceus*, each individual hmw DNA sample used for the ONT library was also used for Illumina library preparation using the Nextera Index Kit (FC-121-1012). For each of *F. catenatus*, *F. nottii*, and *F. xenicus*, Illumina data were multiplexed across two PE150 lanes on an Illumina HiSeq 4000 (RRID:SCR_016386) and demultiplexed by Novogene (Sacramento, CA USA). For *F. olivaceus*, PE150 Illumina NovaSeq reads from one flow cell (2 lanes) were graciously provided by the Texas A&M Agrilife Research Sequencing Facility (College Station, TX USA).

Data Description

Whole genome sequencing data from individuals of four killifish species collected from ONT PromethION (Table 1) and Illumina (NovaSeq and HiSeq 4000) (Table 2) were deposited in the European Nucleotide Archive (ENA) under the study accession PRJEB29136. Deposited raw data are untrimmed and unfiltered. Reads corresponding to lambda phage were filtered from ONT PromethION data using the NanoLyse program from NanoPack (version 1.1.0; [44]). Porechop (RRID:SCR_016967; version 0.2.3) was used to remove residual ONT adapters and NanoFilt (RRID:SCR_016966; version 2.2.0; [44]) was used to filter reads with an average Phred quality score >Q5. After filtering and adapter trimming, ONT data from the PromethION ranged from 30-45x coverage for each species. NanoPlot (version 1.10.0; [44]) was used for visualization of ONT read qualities.

Species	Bases called (Gb)	Cov. (x)	Avg, read length	Reads N50	Q>5 bases called (Gb)	Q>5 avg. read length	ONT signal accession	ONT fastq accession

F. xenicus	38.5	35.0	2,449	5,733; n = 1,373,426	36.42	2,699	ERR3385273	ERR3385269
F. catenatus	40.3	36.6	1,699	3,439; n = 2,687,295	34.28	2,021	ERR3385274	ERR3385270
F. nottii	33.4	30.4	6,480	12,995; n = 700,534	31.06	7,548	ERR3385275	ERR3385271
F. olivaceus	50.1	45.5	4,595	11,670; n = 987,921	45.97	5,365	ERR3385276	ERR3385272

Table 1. ONT data collected from each species. Coverage (cov.) assumes the genome size of each species is 1.1 Gb, as estimated for *F. heteroclitus* [40]. Untrimmed reads were deposited in the ENA under study PRJEB29136. Reads N50 represent the N50 length of all ONT reads before filtering and assembly, followed by the number (n) of reads constituting 50% of the length of all ONT reads. Data used for subsequent genome assemblies were filtered with a requirement for having an average Phred quality score >Q5. The remaining bases called and average read length that are >Q5 are listed.

Average quality scores for all Illumina data were consistently above Q30 (Figure 3A). Residual Nextera adapters and bases with low quality scores were removed from Illumina reads using Trimmomatic PE (version 0.38) with conservative parameters, which included removing bases from each read with a quality score below Q2 and required a minimum read length of 25 bases each [45]. There did not appear to be a difference in the data quality (Figure 3) when the hmwDNA was extracted from flash frozen (*F. olivaceus* and *F. catenaus*) or fresh tissue (*F. xenicus* and *F. nottii*) (Figure 2).

For *F. xenicus* and *F. catenatus*, ONT read qualities ranged from Q5 (minimum cutoff) to Q14 with read lengths generally ranging from 10 bp to 100kb (Figure 3B,C). For *F. nottii and F. olivaceus*, ONT read qualities ranged from Q5 (minimum cutoff) to Q13 with read lengths ranging from 100 bp to 100kb (Figure 3D,E).

**Species**	**Platform**	**Reads (M)**	**Coverage (x)**	**FASTQ Accessions**
F. xenicus	Illumina HiSeq	327.5	89.3	ERR3385278<br>ERR3385279
F. catenatus	Illumina HiSeq	316.5	86.3	ERR3385280<br>ERR3385281
F. nottii	Illumina HiSeq	197.0	53.7	ERR3385282<br>ERR3385283
F. olivaceus	Illumina NovaSeq	601.9	164.0	ERR3385284<br>ERR3385285

Table 2. Illumina data collected were all paired-end 150 reads. Coverage assumes 1.1 Gb genome size measured for *F. heteroclitus* [40].

Draft Assemblies

As a comparison with assemblies using long read ONT data, Illumina data alone were assembled using ABySS version 2.1.5 (RRID:SCR_010709). While the BUSCO scores were consistently above 50% completeness [46], the number of contigs and contig N50 lengths of the Illumina-only assemblies were not acceptable for downstream use (Table 3).

**Species**	**Bases in the Illumina-only assembly**	**N contigs**	**Avg length**	**Largest contig**	**N50**	**Illumina-only BUSCO** C CS/CD/F/M
F. xenicus	1,283,257,056	5,195,861	246.98	71,596	2,571; n = 107,350	57.1% 56.4/0.7/33.3/9.6
F. catenatus	1,205,429,912	3,989,534	302.15	70,870	3,629; n = 80,839	53.8% 52.8/1.0/36.0/10.2
F. nottii	1,167,835,004	3,875,693	301.32	92,540	3,740; n = 72810	62.7% 61.7/1.0/27.4/9.9

F. olivaceus	1,252,948,998	4,509,089	277.87	70,765	3,670; n = 77136	65.7% 64.0/1.7/25.1/9.2

Table 3. Statistics for Illumina-only assemblies using ABySS (version 2.1.5) for each species. The BUSCO Eukaryota database (303 genes) was used to evaluate the completeness of each assembly [46]. BUSCO numbers reported are percentage complete (C) followed by the percentages of complete single-copy (CS), complete duplicated (CD), fragmented (F), missing (M) out of 303 genes.

The ONT-only assemblies using the fuzzy de Bruijn graph assembler, wtdbg2 (RRID:SCR_017225; version 2.3; [47]) had high contig N50 but low complete matches with the BUSCO Eukaryota database (Table 4). The assembler wtdbg2 took an average of 6.1 wall time hours per assembly and required 59 GB RAM. The polishing tool pilon required an average of 65.99 wall time hours and used 1.61 TB RAM. Following polishing with Illumina data using the pilon software tool version 1.23 [33], the BUSCO Eukaryota completeness scores increased to consistently greater than 90% (Table 4). Compared to the BUSCO results for the existing assembly for *F. heteroclitus* (NCBI GCA_000826765.1 Fundulus_heteroclitus-3.0.2) which was 92.4% complete (CS:89.8%,CD:2.6%,F:2.3%,M:5.3%), the BUSCO results for these four species are quite complete. Assemblies were deposited in the Open Science Framework (OSF) repository [48] and zenodo record [49].

**Species**	**Contigs**	**Contig N50**	**Assembly size (bases)**	**Complete BUSCO after wtdbg2 ONT-only**<br>C<br>CS/CD/F/M	**Complete BUSCO after pilon polishing with Illumina**<br>C<br>CS/CD/F/M
F. xenicus	5,621	888,041; n = 325	1,075,031,690	10.2% 10.2/0.0/11.6/78.2	90.5% 87.5/3.0/3.0/6.5
F. catenatus	5,854	436,102; n = 780	1,163,592,740	11.2% 28.4/0.0/24.4/47.2	90.4% 88.4/2.0/2.6/7.0

F. nottii	2,242	2,701,963; n = 95	1,081,276,623	28.4% 11.2/0.0/22.1/66.7	94.4% 92.1/2.3/1.0/4.6
F. olivaceus	2,622	2,669,230; n = 105	1,198,526,423	23.4% 23.4/0.0/25.7/50.9	92.1% 89.8/2.3/1.3/6.6

Table 4. ONT PromethION assemblies using the wtdbg2 version 2.3 assembler [47] followed by polishing with pilon version 1.23 [33]. Of interest is the dramatic improvement of the complete BUSCO metric after polishing with pilon. BUSCO numbers reported are percentage complete (C) followed by the percentages of complete single-copy (CS), complete duplicated (CD), fragmented (F), missing (M) out of the 303 genes in the BUSCO Eukaryota database [46].

Discussion

In this study, we collected 30-45x coverage of ONT data in combination with 50-160x coverage of Illumina PE150 sequencing data and generated draft genome assemblies for four species of *Fundulus* killifish. For the four assemblies presented here, the combination of ONT and Illumina data allowed us to generate highly contiguous assemblies with acceptable BUSCO results. The assemblies generated by ONT data alone were not acceptable for use because of the poor BUSCO results, likely due to the high rate of ONT sequence errors. Polishing the ONT assemblies with Illumina data did not improve contiguity of the assemblies, but served to correct bases, fix mis-assemblies and fill gaps, shown by the large boost in BUSCO scores relative to the ONT assemblies alone. However, even with improved BUSCO scores, assemblies may have high remaining indel rates due to problems inherent in mapping short Illumina reads to repetitive sequences [50].

The Phred base quality scores and the read lengths of the ONT data appeared to make a difference in the contig N50 metrics of the assemblies. Both *F. xenicus* and *F. catenatus* had shorter average read lengths and reads N50 compared to *F. nottii* and *F. olivaceus*. The contig N50 metric for both *F. nottii* and *F. olivaceus* assemblies was larger (>2 Mb) compared to assemblies from *F. xenicus* and *F. catenatus* (<1 Mb). The assembly from *F. nottii*, which had the lowest data yield, had higher average read lengths and higher reads N50 compared to the

other species. *F. olivaceus*, which had the highest yield, also had a higher reads N50 and average read length. Therefore, when generating ONT data for draft genome assemblies, the length and the quality of the reads may matter more than the overall yield of data. This is not easily controlled, except with the quality of the input hmw DNA sample, the quality of the ONT sequencing library and flow cell (Supplemental Figure 1).

We observed lower yields from DNA isolated from our killifish samples compared to similar length DNA isolated from mammalian cultured cell lines. These lower yields are a result of a rapid decline in the active number of pores (Supplemental Figure 1) possibly because of pore blockage. For the sample from *F. olivaceus*, we performed a nuclease flush and re-loaded a second aliquot of the library that helped us improve the yield. Recent improvements in the unblock mechanisms in the MinKnow software along with nuclease flush can help to mitigate the blocking issue. The duty time plot (Supplemental Figure 1) shows 60% pore occupancy at the beginning of the run, which then dropped down to approximately 18% in 17-18 hrs. This was typical of the runs with all of the samples. Through our informal conversations with colleagues this appears to be a known problem in the nanopore community, at least for DNA from marine fish and birds. DNA isolated from these four killifish samples was fragile and easy to degrade as indicated by small fragments below 40 kb in the gel images. We suspect that this fragile DNA as well as pore blockage could be the cause of shorter read lengths and lower yields observed in our runs.

The Vertebrate Genome Project (VGP) lists standards for *de novo* genome assembly that include four types of data: PacBio long reads, 10x Genomics linked Illumina reads, Hi-C chromatin mapping and Bionano Genomics optical maps [51]. Each of these four types of data has associated costs of generation, including analysis and computational time. While chromatin

capture and Hi-C methods produce high quality chromosome-level assemblies [51–54], these data types can significantly increase the overall cost of the genome sequencing project. In this study, we report the pairing of just two data sets: short Illumina reads with long reads from the ONT PromethION platform, to generate a draft assembly at a lower cost. The qualities of the assemblies presented here are not as high compared to the standards recommended by the VGP, which requires the assembly to be haplotype phased with a minimum contig N50 of 1 million bp (1Mb), scaffold N50 of 10Mb, 90% of the genome assembled into chromosomes and a sequence error frequency of at least Q40 [51]. However, the assemblies presented here and for *F. heteroclitus* [40] are sufficient for many uses. For *F. olivaceus* and *F. nottii*, draft assemblies using wtdbg2 [47] and pilon polishing with Illumina data [33] had contig N50 >1 Mb, which meets the minimum requirements for assemblies in downstream synteny analyses [11].

New software tools and methods for base-calling, assembling and analyzing noisy ONT long reads are being developed at a fast rate [55,56]. Because of this fast pace of software tool development for ONT data, standard operating procedures are not available. While we intend to use the four assemblies presented here for comparative evolutionary analyses, the raw data are shared here with the intent that others may use them for tool development and as new workflow pipelines, algorithms, tools, and best practices emerge.

Conclusions

Sequencing data from the ONT PromethION and Illumina platforms combined can contribute to assemblies of eukaryotic vertebrate genomes (>1 Gb). These sequencing data from wild-caught individuals of *Fundulus* killifish species are available for use with tool development and workflow pipelines. Ongoing work from our group is comparing genomic content between

these *Fundulus* species to address questions about evolutionary mechanisms of divergence between marine and freshwater niches.

Data re-use potential

We encourage use and re-use of these data. This collection of whole genome sequencing data from the PromethION and Illumina platforms originates from wild-caught individuals of closely-related *Fundulus* killifish species, obtained for the purpose of comparative evolutionary genomics analyses. These data, which add to the growing set of public data available from the ONT PromethION sequencing platform [25,57], can be used for developing base-calling and assembly algorithms.

Availability of supporting data and materials

Raw data are available in the ENA under study PRJEB29136. Draft assembly data products and quality assessment reports are available in the OSF repository[48] and zenodo [49]. Scripts used for this analysis workflow are available atONT_Illumina_genome_assembly[58]. All supporting data and materials are available in the *GigaScience* GigaDB database [59].

List of abbreviations

BUSCO = Benchmarking Universal Single-Copy Orthologs
ENA = European Nucleotide Archive
hmw DNA = high molecular weight DNA
ONT = Oxford Nanopore Technologies

OSF = Open Science Framework

PE = paired end

VGP = Vertebrate Genome Project

Declarations

Ethical Approval

UC Davis IACUC protocol #17221

Consent for publication

Not applicable.

Competing Interests

The authors declare that they have no competing interests.

Funding

Gordon and Betty Moore Foundation to CTB under award number GBMF4551. IU-TACC Jetstream and PSC Bridges XSEDE allocations TG-BIO160028 and TG-MCB190015 to LKJ.

Author's Contributions

Sample extractions and library preparations were done by LKJ, RS, TG, JR. Project advising by CTB and AW. Manuscript writing and editing by LKJ, RS, TG, JR, LF, CTB, and AW.

Acknowledgements

We thank Dr. David Duvernell at Missouri University of Science & Technology and Dr. Jacob Schaefer at the University of Southern Mississippi for generously collecting and sending fish. A special thank you goes to Dr. Charlie Johnson and Dr. Richard Metz at Texas A&M University

Agrilife Research Sequencing Facility for contributing Illumina NovaSeq data from *Fundulus olivaceus*. Thanks to the instructors and participants at PoreCamp USA (June 2017) for their helpful advice.

Figure Legends

Figure 1. Four *Fundulus* killifish (left to right): the marine diamond killifish *Fundulus xenicus*; the freshwater northern studfish, *Fundulus catenatus* (south central United States); the freshwater bayou topminnow, *Fundulus nottii*; and the freshwater blackspotted topminnow, *Fundulus olivaceus*. (drawings used with permission from the artist, Joseph R. Tomelleri).

Figure 2. Field inversion gels with red boxes showing samples sequenced (in order from left to right: *F. catenatus* (sheared vs. unsheared), *F. olivaceus*, *F. nottii*, *F. xenicus*). DNA was extracted from fresh tissues for *F. xenicus* and *F. nottii*, and from frozen tissues for *F. catenatus* and *F. olivaceus*.

Figure 3. A) Quality score profiles for representative R1 Illumina reads from *F. xenicus*, *F. catenatus*, *F. nottii* (top lines) and *F. olivaceus* (bottom line). For Illumina data, phred quality scores were consistently above Q30 across all reads. Average read quality scores (Q score) vs. read lengths for ONT PromethION from B) *F. xenicus*, C) *F. catenatus*, D) *F. nottii*, E) *F. olivaceus*.

References

1. Mardis E, McPherson J, Martienssen R, Wilson RK, McCombie WR. What is finished, and why does it matter. Genome Res. 2002;12:669–71. http://dx.doi.org/10.1101/gr.032102

2. Baker M. De novo genome assembly: what every biologist should know. Nat Methods; 2012;9:333 https://doi.org/10.1038/nmeth.1935

3. Ekblom R, Wolf JBW. A field guide to whole-genome sequencing, assembly and annotation. Evol Appl [Internet]. 2014;7:1026–42. Available from: http://dx.doi.org/10.1111/eva.12178

4. Stemple DL. So, you want to sequence a genome. Genome Biol [Internet]. 2013;14:128. Available from: http://dx.doi.org/10.1186/gb-2013-14-7-128

5. Li F-W, Harkess A. A guide to sequence your favorite plant genomes. Appl Plant Sci [Internet]. 2018;6:e1030. Available from: http://dx.doi.org/10.1002/aps3.1030

6. Dominguez Del Angel V, Hjerde E, Sterck L, Capella-Gutierrez S, Notredame C, Vinnere Pettersson O, et al. Ten steps to get started in Genome Assembly and Annotation. F1000Res [Internet]. 2018;7. Available from: http://dx.doi.org/10.12688/f1000research.13598.1

7. Ip CLC, Loose M, Tyson JR, de Cesare M, Brown BL, Jain M, et al. MinION Analysis and Reference Consortium: Phase 1 data release and analysis. F1000Res [Internet]. 2015;4:1075. Available from: http://dx.doi.org/10.12688/f1000research.7201.1

8. Tyson JR, O'Neil NJ, Jain M, Olsen HE, Hieter P, Snutch TP. MinION-based long-read sequencing and assembly extends the Caenorhabditis elegans reference genome. Genome Res [Internet]. 2018;28:266–74. Available from: http://dx.doi.org/10.1101/gr.221184.117

9. Ebbert MTW, Jensen TD, Jansen-West K, Sens JP, Reddy JS, Ridge PG, et al. Systematic analysis of dark and camouflaged genes reveals disease-relevant genes hiding in plain sight. Genome Biol [Internet]. 2019;20:97. Available from: http://dx.doi.org/10.1186/s13059-019-1707-2

10. Laver T, Harrison J, O'Neill PA, Moore K, Farbos A, Paszkiewicz K, et al. Assessing the performance of the Oxford Nanopore Technologies MinION. Biomol Detect Quantif [Internet]. 2015;3:1–8. Available from: http://dx.doi.org/10.1016/j.bdq.2015.02.001

11. Liu D, Hunt M, Tsai IJ. Inferring synteny between genome assemblies: a systematic evaluation. BMC Bioinformatics [Internet]. 2018;19:26. Available from: http://dx.doi.org/10.1186/s12859-018-2026-4

12. Tyler AD, Mataseje L, Urfano CJ, Schmidt L, Antonation KS, Mulvey MR, et al. Evaluation of Oxford Nanopore's MinION Sequencing Device for Microbial Whole Genome Sequencing Applications. Sci Rep [Internet]. 2018;8:10931. Available from: http://dx.doi.org/10.1038/s41598-018-29334-5

13. Pfeiffer F, Gröber C, Blank M, Händler K, Beyer M, Schultze JL, et al. Systematic evaluation of error rates and causes in short samples in next-generation sequencing. Sci Rep [Internet]. 2018;8:10950. Available from: http://dx.doi.org/10.1038/s41598-018-29325-6

14. Zeng Y, Martin CH. Oxford Nanopore sequencing in a research-based undergraduate course [Internet]. bioRxiv. 2017 [cited 2019 Jun 20]. p. 227439. Available from: https://www.biorxiv.org/content/10.1101/227439v1

15. Zaaijer S, Columbia University Ubiquitous Genomics 2015 class, Erlich Y. Using mobile sequencers in an academic classroom. Elife [Internet]. 2016;5. Available from: http://dx.doi.org/10.7554/eLife.14258

16. Ducluzeau A-L, Tyson JR, Collins RE, Snutch TP, Hassett BT. Genome Sequencing of Sub-Arctic Mesomycetozoean Sphaeroforma sirkka Strain B5, Performed with the Oxford Nanopore minION and Illumina HiSeq Systems. Microbiol Resour Announc [Internet]. 2018;7. Available from: http://dx.doi.org/10.1128/MRA.00848-18

17. Pomerantz A, Peñafiel N, Arteaga A, Bustamante L, Pichardo F, Coloma LA, et al. Real-time DNA barcoding in a rainforest using nanopore sequencing: opportunities for rapid biodiversity assessments and local capacity building. Gigascience [Internet]. 2018;7. Available from: http://dx.doi.org/10.1093/gigascience/giy033

18. Boykin LM, Ghalab A, De Marchi BR, Savill A, Wainaina JM, Kinene T, et al. Real time portable genome sequencing for global food security [Internet]. bioRxiv. 2018 [cited 2019 Jun 20]. p. 314526. Available from: https://www.biorxiv.org/content/10.1101/314526v2

19. Quick J, Loman NJ, Duraffour S, Simpson JT, Severi E, Cowley L, et al. Real-time, portable genome sequencing for Ebola surveillance. Nature [Internet]. 2016;530:228–32. Available from: http://dx.doi.org/10.1038/nature16996

20. Quick J, Grubaugh ND, Pullan ST, Claro IM, Smith AD, Gangavarapu K, et al. Multiplex PCR method for MinION and Illumina sequencing of Zika and other virus genomes directly from clinical samples. Nat Protoc [Internet]. 2017;12:1261–76. Available from: http://dx.doi.org/10.1038/nprot.2017.066

21. Kafetzopoulou LE, Pullan ST, Lemey P, Suchard MA, Ehichioya DU, Pahlmann M, et al. Metagenomic sequencing at the epicenter of the Nigeria 2018 Lassa fever outbreak. Science [Internet]. 2019;363:74–7. Available from: http://dx.doi.org/10.1126/science.aau9343

22. Schalamun M, Nagar R, Kainer D, Beavan E, Eccles D, Rathjen JP, et al. Harnessing the MinION: An example of how to establish long-read sequencing in a laboratory using challenging plant tissue from Eucalyptus pauciflora. Mol Ecol Resour [Internet]. 2019;19:77–89. Available from: http://dx.doi.org/10.1111/1755-0998.12938

23. Jain M, Koren S, Miga KH, et al. Nanopore sequencing and assembly of a human genome with ultra-long reads. Nat Biotechnol. 2018;36(4):338- 345. doi:10.1038/nbt.4060

24. Schmidt MH-W, Vogel A, Denton AK, Istace B, Wormit A, van de Geest H, et al. De Novo Assembly of a New Solanum pennellii Accession Using Nanopore Sequencing. Plant Cell [Internet]. 2017;29:2336–48. Available from: http://dx.doi.org/10.1105/tpc.17.00521

25. De Coster W, De Rijk P, De Roeck A, et al. Structural variants identified by Oxford Nanopore PromethION sequencing of the human genome. Genome Res. 2019;29(7):1178- 1187. doi:10.1101/gr.244939.118

26. Kim HS, Jeon S, Kim C, et al. Chromosome-scale assembly comparison of the Korean Reference Genome KOREF from PromethION and PacBio with Hi-C mapping information. Gigascience. 2019;8(12):giz125. doi:10.1093/gigascience/giz125

27. Tan MH, Austin CM, Hammer MP, Lee YP, Croft LJ, Gan HM. Finding Nemo: hybrid assembly with Oxford Nanopore and Illumina reads greatly improves the clownfish (*Amphiprion ocellaris*) genome assembly. Gigascience. 2018;7:1–6. http://dx.doi.org/10.1093/gigascience/gix137

28. Miller DE, Staber C, Zeitlinger J, Hawley RS. Highly Contiguous Genome Assemblies of 15 Drosophila Species Generated Using Nanopore Sequencing. G3 [Internet]. 2018;8:3131–41. Available from: http://dx.doi.org/10.1534/g3.118.200160

29. Cao MD, Nguyen SH, Ganesamoorthy D, Elliott AG, Cooper MA, Coin LJ. Scaffolding and completing genome assemblies in real-time with nanopore sequencing. Nat Commun. 2017;8:14515. Published 2017 Feb 20. doi:10.1038/ncomms14515

30. Giordano F, Aigrain L, Quail MA, Coupland P, Bonfield JK, Davies RM, et al. De novo yeast genome assemblies from MinION, PacBio and MiSeq platforms. Sci Rep [Internet]. 2017;7:3935. Available from: http://dx.doi.org/10.1038/s41598-017-03996-z

31. Austin CM, Tan MH, Harrisson KA, Lee YP, Croft LJ, Sunnucks P, et al. De novo genome assembly and annotation of Australia's largest freshwater fish, the Murray cod (Maccullochella peelii), from Illumina and Nanopore sequencing read. Gigascience [Internet]. 2017;6:1–6. Available from: http://dx.doi.org/10.1093/gigascience/gix063

32. Vaser R, Sović I, Nagarajan N, Šikić M. Fast and accurate de novo genome assembly from long uncorrected reads. Genome Res [Internet]. 2017;27:737–46. Available from: http://dx.doi.org/10.1101/gr.214270.116

33. Walker BJ, Abeel T, Shea T, Priest M, Abouelliel A, Sakthikumar S, et al. Pilon: an integrated tool for comprehensive microbial variant detection and genome assembly improvement. PLoS One [Internet]. 2014;9:e112963. Available from: http://dx.doi.org/10.1371/journal.pone.0112963

34. Whitehead A. The evolutionary radiation of diverse osmotolerant physiologies in killifish (Fundulus sp.). Evolution [Internet]. 2010;64:2070–85. Available from: http://dx.doi.org/10.1111/j.1558-5646.2010.00957.x

35. Griffith RW. Environment and Salinity Tolerance in the Genus Fundulus. Copeia [Internet]. [American Society of Ichthyologists and Herpetologists (ASIH), Allen Press]; 1974;1974:319–31. Available from: http://www.jstor.org/stable/1442526

36. Durack PJ, Wijffels SE, Matear RJ. Ocean salinities reveal strong global water cycle intensification during 1950 to 2000. Science [Internet]. 2012;336:455–8. Available from: http://dx.doi.org/10.1126/science.1212222

37. Burnett KG, Bain LJ, Baldwin WS, Callard GV, Cohen S, Di Giulio RT, et al. Fundulus as the premier teleost model in environmental biology: opportunities for new insights using genomics. Comp Biochem Physiol Part D Genomics Proteomics [Internet]. 2007;2:257–86. Available from: http://dx.doi.org/10.1016/j.cbd.2007.09.001

38. Reid NM, Proestou DA, Clark BW, Warren WC, Colbourne JK, Shaw JR, et al. The genomic landscape of rapid repeated evolutionary adaptation to toxic pollution in wild fish. Science [Internet]. 2016;354:1305–8. Available from: http://dx.doi.org/10.1126/science.aah4993

39. Oziolor EM, Reid NM, Yair S, Lee KM, Guberman VerPloeg S, Bruns PC, et al. Adaptive introgression enables evolutionary rescue from extreme environmental pollution. Science [Internet]. 2019;364:455–7. Available from: http://dx.doi.org/10.1126/science.aav4155

40. Reid NM, Jackson CE, Gilbert D, Minx P, Montague MJ, Hampton TH, et al. The landscape of extreme genomic variation in the highly adaptable Atlantic killifish. Genome Biol Evol [Internet]. 2017; Available from: http://dx.doi.org/10.1093/gbe/evx023

41. Ghedotti MJ, Davis MP. Phylogeny, Classification, and Evolution of Salinity Tolerance of the North American Topminnows and Killifishes, Family Fundulidae (Teleostei: Cyprinodontiformes). Fieldiana Life Earth Sci [Internet]. 2013;7:1–65. Available from: http://www.bioone.org/doi/abs/10.3158/2158-5520-12.7.1

42. Quick J. Ultra-long read sequencing protocol for RAD004 [Internet]. protocols.io; 2018 [cited 2019 Jun 20]. Available from: https://www.protocols.io/view/ultra-long-read-sequencing-protocol-for-rad004-mrxc57n

43. Wasko AP, Martins C, Oliveira C, Foresti F. Non-destructive genetic sampling in fish. An improved method for DNA extraction from fish fins and scales. Hereditas [Internet]. 2003;138:161–5. Available from: http://dx.doi.org/10.1034/j.1601-5223.2003.01503.x

44. De Coster W, D'Hert S, Schultz DT, Cruts M, Van Broeckhoven C. NanoPack: visualizing and processing long-read sequencing data. Bioinformatics [Internet]. 2018;34:2666–9. Available from: http://dx.doi.org/10.1093/bioinformatics/bty149

45. MacManes MD. On the optimal trimming of high-throughput mRNA sequence data. Front Genet [Internet]. 2014;5:13. Available from: http://dx.doi.org/10.3389/fgene.2014.00013

46. Simão FA, Waterhouse RM, Ioannidis P, Kriventseva EV, Zdobnov EM. BUSCO: assessing genome assembly and annotation completeness with single-copy orthologs. Bioinformatics [Internet]. 2015;31:3210–2. Available from: http://dx.doi.org/10.1093/bioinformatics/btv351

47. Ruan J, Li H. Fast and accurate long-read assembly with wtdbg2 [Internet]. bioRxiv. 2019 [cited 2019 Jun 20]. p. 530972. Available from: https://www.biorxiv.org/content/10.1101/530972v1

48. OSF repository. https://doi.org/10.17605/osf.io/zjv86

49. zenodo. https://doi.org/10.5281/zenodo.3251033

50. Watson, M. and Warr, A. Errors in long-read assemblies can critically affect protein prediction. Nature Biotech. 2019; 37:124-128.

51. Vertebrate Genome Project. A reference standard for genome biology. Nat Biotechnol. 2018;36:1121. http://dx.doi.org/10.1038/nbt.4318

52. Olsen R-A, Bunikis I, Tiukova I, Holmberg K, Lötstedt B, Pettersson OV, et al. De novo assembly of Dekkera bruxellensis: a multi technology approach using short and long-read sequencing and optical mapping. Gigascience. 2015;4:56. http://dx.doi.org/10.1186/s13742-015-0094-1

53. Bickhart DM, Rosen BD, Koren S, Sayre BL, Hastie AR, Chan S, et al. Single-molecule sequencing and chromatin conformation capture enable de novo reference assembly of the domestic goat genome. Nat Genet. 2017;49:643–50. http://dx.doi.org/10.1038/ng.3802

54. Belser C, Istace B, Denis E, Dubarry M, Baurens F-C, Falentin C, et al. Chromosome-scale assemblies of plant genomes using nanopore long reads and optical maps. Nat Plants. 2018;4:879–87. http://dx.doi.org/10.1038/s41477-018-0289-4

55. Low WY, Tearle R, Bickhart DM, Rosen BD, Kingan SB, Swale T, et al. Chromosome-level assembly of the water buffalo genome surpasses human and goat genomes in sequence contiguity. Nat Commun. 2019;10:260. http://dx.doi.org/10.1038/s41467-018-08260-0

56. de Lannoy C, de Ridder D, Risse J. The long reads ahead: *de novo* genome assembly using the MinION. F1000Res. 2017;6:1083. http://dx.doi.org/10.12688/f1000research.12012.2

57. Cali DS, Kim JS, Ghose S, Alkan C, Mutlu O. Nanopore Sequencing Technology and Tools for Genome Assembly: Computational Analysis of the Current State, Bottlenecks and Future Directions. arXiv [q-bio.GN]. 2017. http://arxiv.org/abs/1711.08774

58. Johnson, L. K. 2019a. Dib-lab/ONT_Illumina_genome_assembly (version 2.0). https://doi.org/10.5281/zenodo.3492222.

59. Johnson LK; Sahasrabudhe R; Gill JA; Roach JL; Froenick L; Brown CT; Whitehead A (2020): Supporting data for "Draft genome assemblies using sequencing reads from Oxford Nanopore Technology and Illumina platforms for four species of North American Fundulus killifish" *GigaScience* Database. http://dx.doi.org/10.5524/100747

Figure 1

Click here to access/download;Figure;Figure1.jpg

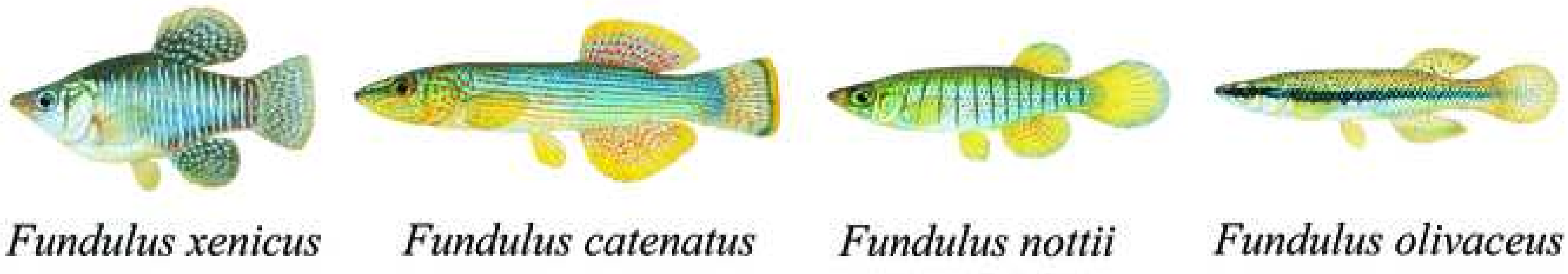

Figure 2

Click here to access/download;Figure;Figure2.png

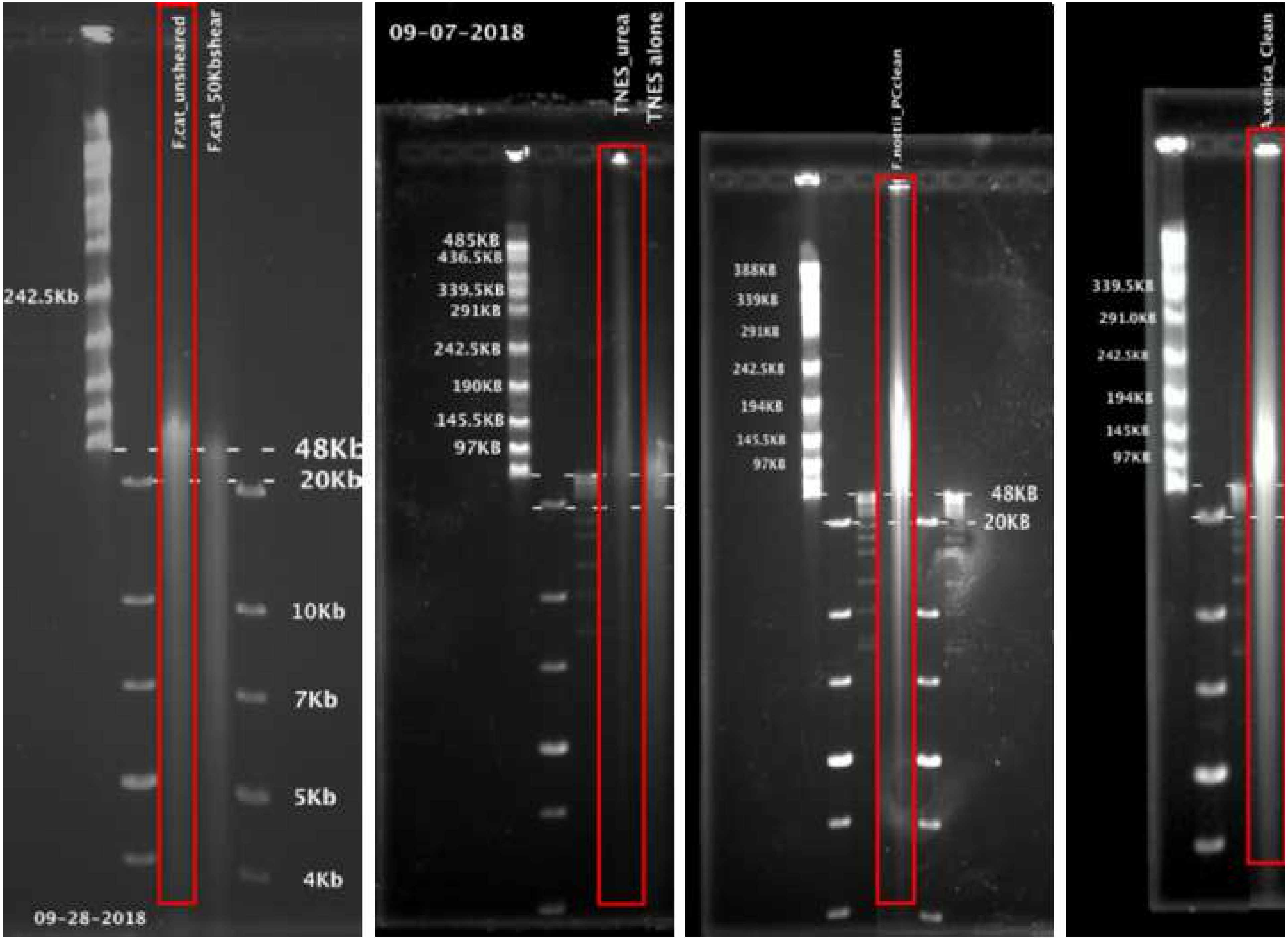

Figure 3

Click here to access/download;Figure;Figure3.jpg

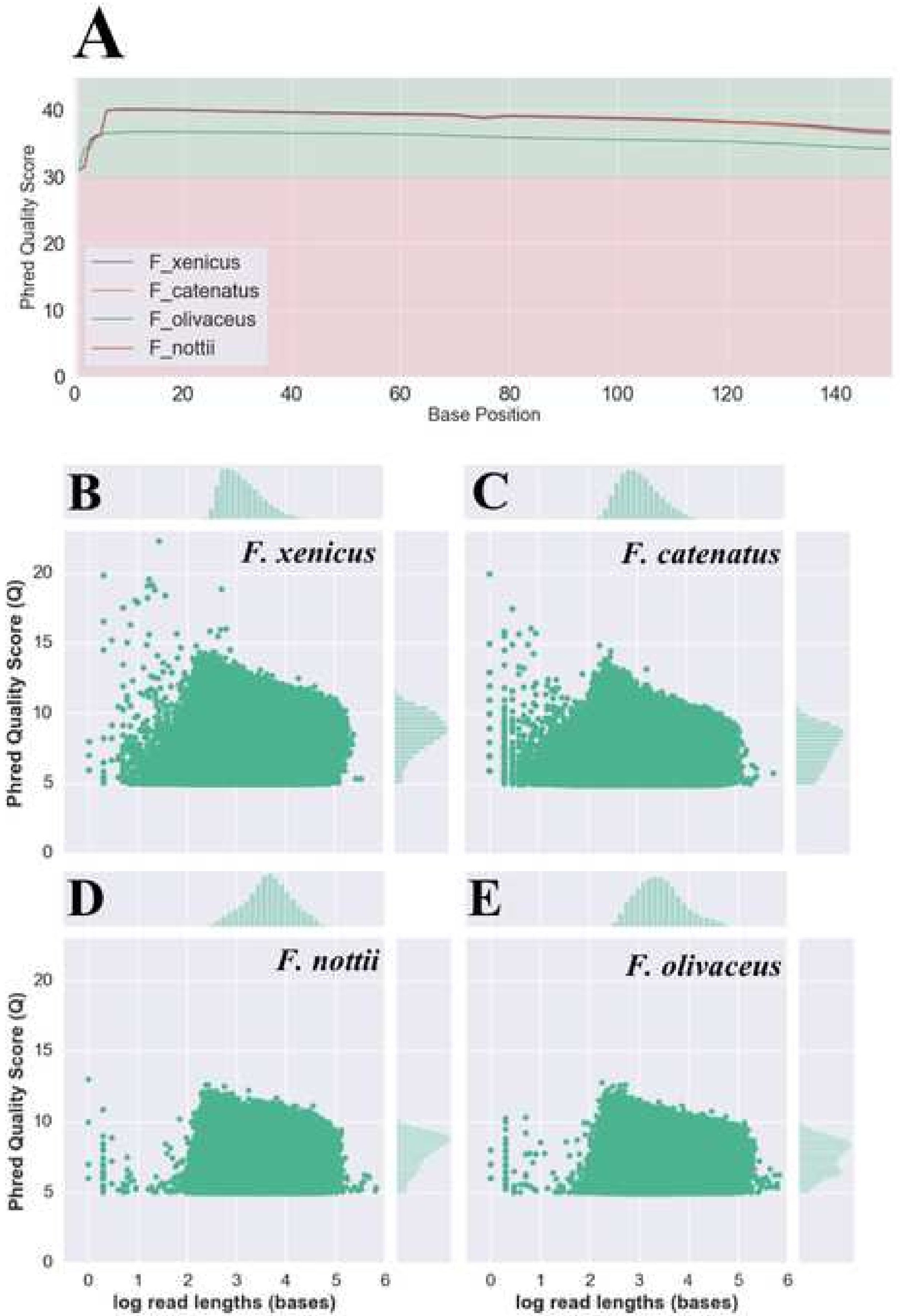

Click here to access/download
Supplementary Material
Johnsonetal_4killifishgenomes_SupplementalInformation_AW.docx
