# Supplementary material for: Draft genome assemblies using sequencing reads from Oxford Nanopore Technology and Illumina platforms for four species of North American Fundulus killifish
Source: Gigascience. 2020 Jun 18;9(6):giaa067. doi: 10.1093/gigascience/giaa067 (PMC7301629; doi:10.1093/gigascience/giaa067)
Supplement: giaa067_Supplemental_File [file giaa067_supplemental_file.docx]

**Supplemental Information**

*Draft genome assemblies using sequence reads from Oxford Nanopore Technologies and Illumina platforms for four species of North American Fundulus killifish*

Supplemental Table 1. Flow cell and basecaller version summary for all the PromethION sequencing runs.

| **Sample name** | **Flow Cell type** | **Flow Cell ID** | **MinKNOW version** | **Basecaller version** |
| --- | --- | --- | --- | --- |
| *F. xenicius* | PRO001 (R9.41) | PAC22364 | v2.0 | ont-guppy v1.4.3 |
| *F. nottii* | PRO002 (R9.4.1) | PAD00901 | v2.1 | ont-guppy v1.6.0 |
| *F. catenatus* | PRO002 (R9.4.1) | PAD11611 | v2.2 | ont-guppy v1.8.1 |
| *F. olivaceus* | PRO002 (R9.4.1) | PAD11766 | v2.2 | ont-guppy v1.8.1 |


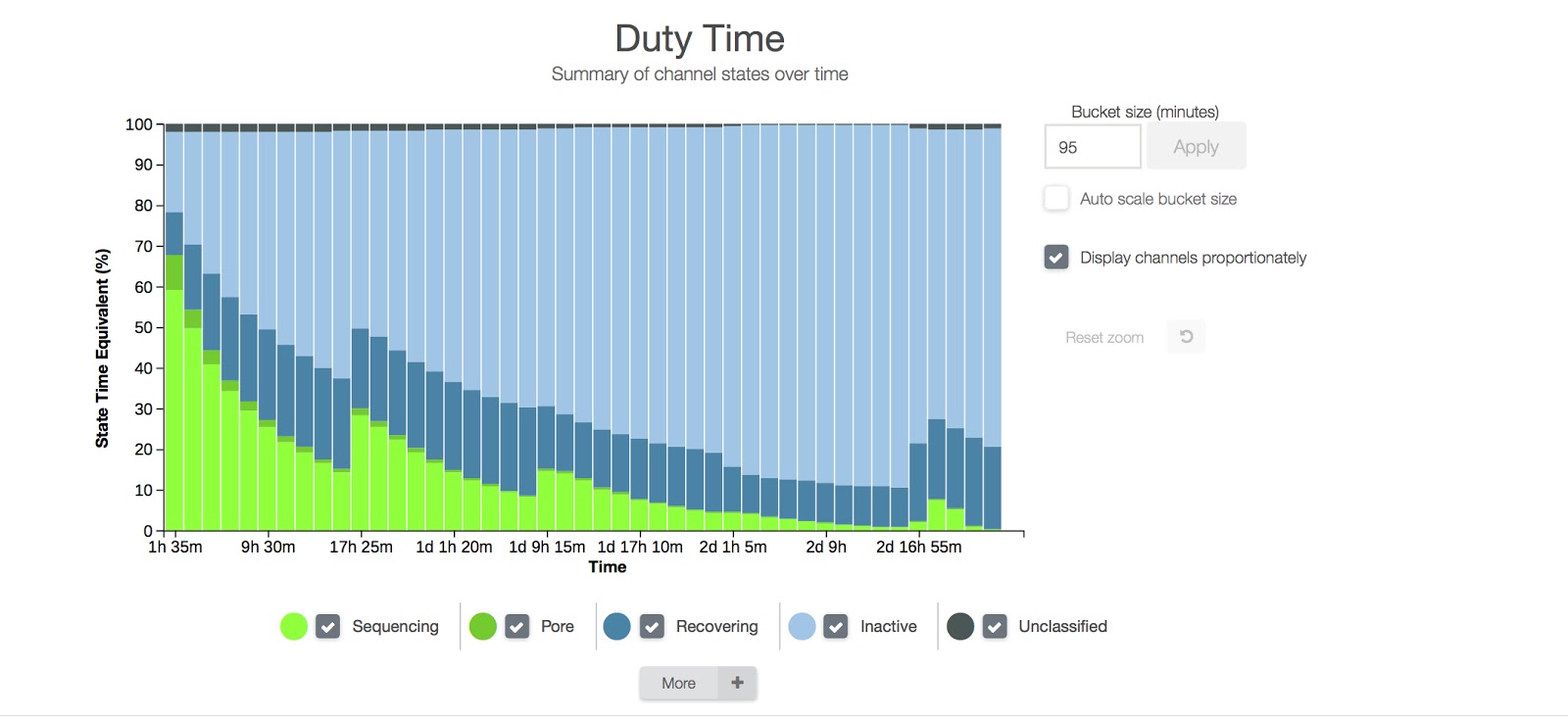


Supplemental Figure 1. Duty time plot of the *F. nottii* sequencing run on PromethION indicating rapid decline in the number of active pores. This plot was typical of all samples. The pore occupancy started at 60% then dropped down to less than 20% in 17 hrs.
