# Supplementary material for: Draft genome assemblies using sequencing reads from Oxford Nanopore Technology and Illumina platforms for four species of North American Fundulus killifish
Source: Gigascience. 2020 Jun 18;9(6):giaa067. doi: 10.1093/gigascience/giaa067 (PMC7301629; doi:10.1093/gigascience/giaa067)
Supplement: giaa067_Response_to_Reviewer_Comments_Original_Submission [file giaa067_response_to_reviewer_comments_original_submission.pdf]

Author's Response To Reviewer Comments

Close

We thank the reviewers for their time and for providing these comments, which have improved this Data Note paper.

Reviewer #1:

>The authors from this paper "Draft genome assemblies using sequencing reads from Oxford Nanopore Technology and Illumina platforms for four species of North American killifish from the Fundulus genus" has reported four species of famous Fundulus killifish assembled by the combination of ONT data and Illumina data. The quality of these four assemblies was pretty well. Their BUSCO results also showed high completeness. The assembling pipeline was described in detail.
>However, the language of this article should be gone through carefully. Too much errors were in the manuscript. As well as, only assembly data were really limited.

Thank you for your comments on our 4 killifish genome assemblies using ONT and Illumina sequencing data. We have fixed the errors you have found; thank you for bringing them to our attention. We have carefully revised the language of the paper according to your recommendations.

>For the abstract: the format should be revised for gigascience publication.

Done. As specified by the GigaScience Data Note instructions for text formatting, we have separated the abstract into three separate sections: Background, Findings, and Conclusions

>Key words: "genome assembly" should be genome assemblies.

We have changed this keyword to “genome assemblies”

>Lines 32-35, this sentence is too heavy, please split it to be clearer.

We have changed this sentence from:

Single-molecule long read nucleic acid sequencing technology from Oxford Nanopore Technologies (ONT), which has been commercially available since 2014 [7], has been shown to improve the contiguity of reference assemblies [8] and reveal “dark regions” that were previously camouflaging genes [9].

to

Single-molecule long read nucleic acid sequencing technology from Oxford Nanopore Technologies (ONT) has been commercially available since 2014 [7]. This technology has been shown to improve the contiguity of reference assemblies [8] and reveal “dark regions” that were previously camouflaging genes [9].

>Line 56, remove a "genome" from "genome genome assembly".

We have removed this redundant word.

>The decimal place of number should be uniform through whole article, 91.2 Gb in line 50, 110.96 Gb in line 52 and 70.6 Gb in 63 should be changed.

We changed the number 110.96 to 111.0 Gb.

>Line 72, "after polishing" should be changed to "after being polished".

We have made this correction.

>Line 77, how about "repeated genomic divergence". It means multiple genomic divergence?

We have changed this sentence from:

Fundulus is a comparative evolutionary model system 76 for studying repeated genomic divergence between marine and freshwater species. Fundulus 77 killifish have a cosmopolitan geographic distribution across North America.

to

Fundulus is a comparative model system for studying genomic divergence that has repeated independently multiple times throughout evolutionary history between marine and freshwater species.

>Line 91, what's the real meaning of "the functional basis of, and evolution of,"?

We have removed this sentence since it is not relevant to the manuscript. Readers who would like more information about this statement are invited to see the citations listed.

The manuscript now reads:

The Atlantic killifish, Fundulus heteroclitus has been a well-described model organism for investigating physiological resilience to temperature, salinity, hypoxia, and environmental pollution [34,37–39]. There is a reference genome available for F. heteroclitus [40]. However, we do not currently have any reference genomes from other Fundulus killifish, particularly from those occupying freshwater habitats.

>For the Figure 1, the quality of this figure is too poor, please revise.

We apologize for the low quality resolution in Figure 1. Thank you for bringing this to our attention. High resolution images will be provided to the publisher.

>Line 128, for the "both the ultra-long read sequencing protocol from [42]", "from" what?

We have changed this sentence from:

For 127 F. catenatus and F. olivaceus, both the ultra-long read sequencing protocol from [42] (which 128 included Tissue lysis buffer with Tris, NaCl, EDTA, SDS and Proteinase K followed by 129 phenol:chloroform extraction), as well as the Qiagen "DNA purification from tissue using the 130 Gentra puregene Tissue Kit" (p. 39) were used, and were found to be similar to the Agilent kit.

to

For F. catenatus and F. olivaceus, two extraction methods were tested: 1) Tris, NaCl, EDTA, SDS and Proteinase K followed by phenol:chloroform extraction [42] and 2) the Qiagen "DNA purification from tissue using the Gentra puregene Tissue Kit" (p. 39).

>Line 142, "tissue" should tissues.

We have made this correction.

>Line 150, "reads N50" should be changed to "read N50".

We feel that we need to keep this wording to make the distinction between ONT reads N50 as opposed to genome assembly N50.

>Only the genome assemblies are indeed limited for further using, like comparative genomic analysis. This will seriously impede the power of this important article and these famous species. I strongly suggest the authors to perform the genome annotation of these four killifish, and re-submit this article.

Thank you for this suggestion. We agree that annotations will be a valuable resource for these genomes and for comparative analyses. However, generating accurate and reliable annotations for new genome assemblies requires considerable additional work. Given the usefulness of these new genome assemblies

and raw PromethION sequencing data to the broader research community, we chose to contribute a Data Note, which makes these data immediately accessible. An annotation effort is ongoing by our research group and others are welcome to also pursue annotation.

Reviewer #2:

We thank Reviewer #2 for their comments and observation that the data are accessible online.

>As is appropriate for a Data Note, the manuscript lacks the analysis that is expected of a genomes paper. It does not present coverage analysis, which could indicate areas of mis-assembly. It does not include the gene annotation of any assembly. It does not include any alignments between the four new assemblies or the previously published one. It does not speculate on possible causes of the six-fold variation in contig N50.

Yes, this is accurate. The manuscript does lack the analysis expected of a genome paper, and does not present mis-assembly coverage analysis, annotations, comparative genomic analyses, or assembly quality analysis beyond BUSCO scores.

>1. The tables and figures could use some adjustment to increase their value to readers.
>The caption to Table 1 should explain or reference the N50 vs n statistics in column 5, and the Q>5 threshold in columns 6 and 7. The caption to Figure 3 should label the five colored wiggle plots that appear in 3A. Table 2 should use significant digits consistently. Table 3 should list the species in the same order as the other tables for ease of comparison.

Thank you for bringing this to our attention. We made the following adjustments to the tables and figures:

• We added this to the caption to Table 1:

Reads N50 represent the N50 length of all ONT reads before filtering and assembly; followed by the number (n) of reads constituting 50% of the length of all ONT reads. Data used for subsequent genome assemblies were filtered with a requirement for having an average Phred quality score >Q5). The remaining bases called and average read length that are >Q5 are listed.

• The Q vs. cycle plots in Figure 3A were adjusted to only include R1, for ease of labeling (reducing the number of lines from 14 down to 4 and a figure legend was added to indicate which lines correspond to which species. For full details of this plot, please see this notebook:
https://github.com/dib-lab/ONT_Illumina_genome_assembly/blob/master/qc/fastqc/plots_all_fadapa.ipynb

• We fixed the significant digits in Table 2 and changed order of species in all tables so that they are consistent.

>2. The discussion of cost should be more disciplined. This paper refers to "the low cost" of its assemblies in contrast to "high cost of generation" with other technologies. However, no cost data is presented or referenced. Prices are difficult to pin down but there must be some way to provide a helpful comparison backed by facts.

We agree that sequencing prices are difficult to pin down, e.g. what we paid might be different than another research group with access to different facilities. We edited the wording to generally discuss costs of data types, rather than specific costs. This paragraph now reads:

The Vertebrate Genome Project (VGP 2018) lists standards for de novo genome assembly that include four types of data: PacBio long reads, 10x linked Illumina reads, Hi-C chromatin mapping and Bionano Genomics optical maps. Each of these four types of data has associated costs of generation, including analysis and computational time. While chromatin capture and Hi-C methods produce high quality chromosome-level assemblies [48–51], these data types can significantly increase the overall cost of the genome sequencing project. Here, we report the pairing of high-accuracy short Illumina reads with error-prone long reads from the ONT PromethION platform (two out of the four VGP required sequencing datasets) to generate a draft assembly at a minimum cost. The qualities of these assemblies are not as high as compared to the standards recommended by VGP (2018) with the 3.4.2.QV40 phased metric, which requires the assembly to be haplotype phased with a minimum contig N50 of 1 million bp (1Mb),

scaffold N50 of 10Mb, 90% of the genome assembled into chromosomes and a base quality error of Q40, (VGP 2019). However, for many research purposes these assemblies are sufficient, considering that we have a high-quality reference genome assembly for another species within the genus [40]. For F. olivaceus and F. nottii, draft assemblies using wtdbg2 [47] and pilon polishing with Illumina data [33] had contig N50 >1 Mb, which meets the minimum requirements for assemblies in downstream synteny analyses [11].

>3. The title names two companies. Could it instead refer to polished nanopore assemblies?

We think that a title that specifically names the sequencing technologies will help readers to find the paper and accordingly would like to keep the current title.

We have added the word “polish” to the keywords and in the abstract, to highlight the importance of the polishing analysis on improving the quality of the genome assemblies.

>4. The discussion section should be revised to remove imprecise wording.
>* Quality standards of the VGP could use better explanation and citation.

We inserted a better citation for the Vertebrate Genome Project (2018):

Vertebrate Genome Project. A reference standard for genome biology. Nat Biotechnol. 2018;36:1121. Available from: http://dx.doi.org/10.1038/nbt.4318

>* The phrase "these assemblies" is ambiguous in places.

We changed the wording to: “For the four assemblies presented here”

>* Pilon "served to correct errors"; which kind?

We changed the wording to "served to correct bases, fix mis-assemblies and fill gaps", to be more specific about the corrections made with the Pilon program.

>* The "qualities of the ONT data appeared to make a difference"; which qualities?

We clarified the wording in this paragraph. We changed from:

The qualities of the ONT data appeared to make a difference in the contig N50 metrics of the assemblies. F. nottii and F. olivaceus both had contig N50 >2Mb, while assemblies from F. xenicus and F. catenatus had contig N50 <1Mb. F. xenicus and F. catenatus had shorter average read lengths and reads N50, on average, compared to F. nottii and F. olivaceus. F. nottii, which had the lowest yield, had higher average read lengths and reads N50 compared to the other species. F. olivaceus, which had the highest yield, also had a high reads N50 and average read length. Therefore, when generating ONT data for draft genome assemblies, it might matter more to have a lower yield of longer reads than a higher yield of shorter reads.

to:

The Phred base quality scores and the read lengths of the ONT data appeared to make a difference in the contig N50 metrics of the assemblies. Both F. xenicus and F. catenatus had shorter average read lengths and reads N50 compared to F. nottii and F. olivaceus. The contig N50 metric for both F. nottii and F. olivaceus was larger (>2 Mb) compared to F. xenicus and F. catenatus (<1 Mb). F. nottii, which had the lowest data yield, had higher average read lengths and higher reads N50 compared to the other species. F. olivaceus, which had the highest yield, also had a high reads N50 and average read length. Therefore, when generating ONT data for draft genome assemblies, the size and the quality of the reads matter more than the overall yield of data. This can be input quality of the DNA sample or the quality of the flow cell.

>* "It might matter more to have lower yield of longer reads"; matter how? Anyway, is that a setting on the sequencer?

Please see the above change to this paragraph. We clarified by mentioning that the combination of the quality of the hmw DNA sample and the ONT library seem to make more of a difference for the read

length and quality than the overall yield of data.

>* "Our samples were consistently not using pores as efficiently" seems to be a colloquialism.

We changed the wording to:

We observed lower yields from DNA isolated from our killifish samples compared to similar length DNA isolated from mammalian cultured cell lines. These lower yields are a result of a rapid decline in the active number of pores (Supplemental Figure 1) possibly because of pore blockage. For the sample from F. olivaceus, we performed a nuclease flush and re-loaded a second aliquot of the library that helped us improve the yield. Recent improvements in the unblock mechanisms in the MinKnow software along with nuclease flush can help to mitigate the blocking issue. The duty time plot (Supplemental Figure 1) shows 60% pore occupancy at the beginning of the run, which then dropped down to approximately 18% in 17-18 hrs. This was typical of the runs with all of the samples. Through our informal conversations with colleagues this appears to be a known problem in the nanopore community, at least for DNA from marine fish and birds.

>* A "brittle property" of the DNA is probably speculation.

DNA isolated from these four killifish samples was fragile and easy to degrade as indicated by small fragments below 40 kb in the gel images. This fragile DNA as well as the known ability of fish DNA to block pores could be the cause of shorter read lengths and lower yields observed in our runs. We changed our wording to “DNA isolated from these four killifish samples was fragile and easy to degrade as indicated by small fragments below 40 kb in the gel images. We suspect that this fragile DNA as well as pore blockage could be the cause of shorter read lengths and lower yields observed in our runs.”

>* One paper's recommendation for synteny analysis may not warrant being called THE minimum requirements.

We clarified the wording to specifically mention synteny analysis and not overemphasize meeting all minimum requirements.

Reviewer #3:

>This is a useful study describing the sequencing and initial assembly of four Fundulus species. Although there is a lot of interesting genome biology to be discovered, this paper focuses on the technicalities of data collection only.

Thank you, we are glad you find this study useful. Yes, it is true that we have focused on the technical aspects of generating the data and the assemblies.

>To be useful for the intended benchmarking purposes, I would like to ask the authors to supply some additional details on several stages of the sequencing and analyses:
>P3, Methods and Results: How did you identify the species?

Fish samples were identified in the field by experts. We clarified by adding the following wording:

Live field-caught individuals of each fish species were identified by field experts, shipped to UC Davis and kept at their native salinities in an animal holding facility maintained according to University of California IACUC standards.

>P5, ONT sequencing: I assume you used R9.4(.1?) flowcells. Please list the exact types, as these have changed several times already and are about to change again. Also, which version of which ONT basecaller did you use?

The chart with flow cell and basecaller version summary for all the runs was added as Supplemental Table 1.

>P6, Table 1: Coverage is calculated using a reference species genome, but it should be straightforward to estimate it for each species based on Illumina k-mer profiles. The assembly sizes (Tables 3,4) vary by ~10%, it would be nice to know whether this reflects actual differences in genome size between the species. In addition, this will yield an estimate of heterozygosity, which is relevant for the interpretation

of the Illumina assemblies (high heterozygosity may result in harder to assemble genomes when using short reads).

Thank you for the suggestion. We agree that an assembly-independent genome size estimation would be valuable and it would be nice to know whether the genome size differences in Tables 3,4 are due to actual differences between species or because of assembly errors. This is actually a non-trivial analysis requiring the use of additional software tools and computing resources. Given the usefulness of these new genome assemblies and the raw PromethION sequencing data to the broader research community, we chose to contribute a Data Note, which makes these data immediately accessible. Future genome size estimation could be part of future analysis using these genomes.

>P8/9, BUSCO scores: It would be very informative if you included a positive control here, by running BUSCO on the F. heteroclitus reference genome. (PS I am somewhat surprised the fragmented Illumina assemblies yield relatively decent completeness scores. Could this be a result of the specific reference database (eukaryotes) used? Perhaps strongly conserved genes are often found in regions of the genome that are relatively easy to assemble?)

Thank you for this suggestion. We performed the positive control BUSCO run and added the following:

Comparing to the BUSCO results for the existing assembly for sister F. heteroclitus (NCBI GCA_000826765.1 Fundulus_heteroclitus-3.0.2), which was 92.4% complete (CS:89.8%,CD:2.6%,F:2.3%,M:5.3%), the BUSCO results for these four species are quite complete.

>P9, l221: How did you run Pilon? Some recommend running it multiple times? Polishing will have changed the genome lengths/N50s listed in Table 4.

We ran several iterations of the pilon program to polish assemblies, as was recommended by the methods used in Miller et al. (2018): https://www.ncbi.nlm.nih.gov/pubmed/30087105 We found that these extra iterations of pilon did not change the quality of the assemblies so we have reported only one iteration with pilon. In general, the lengths of the contigs and the N50 scores did not change with pilon because it is correcting individual bases and not changing the continuity of the contigs.

>P10, Discussion: There is some variation in sample storage conditions, DNA isolation, library prep methods, etc. As far as I can see, there are no clear correlations with data and assembly quality, but perhaps you could summarize this here?

The following was added to summarize:

There did not appear to be a difference in the data quality (Figure 3) when the hmwDNA was extracted from fresh frozen (F. olivaceus and F. catenaus) or fresh tissue (F. xenicus and F. nottii) (Figure 2).

Some minor thoughts & comments:

>P2, l49: Although PromethION has higher throughput than MinION, this section also compares ONT data from different timepoints. Changes in chemistry and protocols are probably of greater influence on the increases in throughput than the platform. Today, MinION throughput is (under good conditions) higher than the 6.5 Gbp mentioned.

Yes, the improvements with pore chemistry and protocols are increasing the yield with MinION. However, the pore chemistry is inherently different in the PromethION because of the higher density of pores on the flow cells. We have added this to the end of the paragraph to clarify:

While changes in pore chemistry and protocols are improving the yields from the ONT MinION, the yield from the ONT PromethION platform remains higher because of the higher density of nanopore channels.

>P2, l67, 'Recently…': This approach using Illumina polishing is not of a more recent date than the scaffolding method. Fish genomes produced using either method were published in 2017 already.

We removed the word "recently" from this sentence.

>P3, l89: This sentence introduces the Atlantic killifish twice.

This redundancy was removed.

P9, l220: I assume the 6.1 hours for wtdbg2 are not CPU hours (as listed for Pilon, next line)? I have used it to assemble a similar genome, which took ~350 CPU hours.

Yes, we clarified that the hours reported are wall time hours, not CPU hours.

>P10, Discussion, third paragraph: DNA from fatty fish is notoriously difficult to sequence, with low yields due to rapid pore deaths. I think the cause remains unknown (and the phenomenon unpublished?). Although the fish sequenced here are not that rich in oils, did you observe the same phenomenon (i.e. when you mention 'not using pores as efficiently')?

Yes, the cause of low yields due to rapid pore deaths remains unknown. We have searched the literature and have not seen anything related to this, so felt that it was important to document here what we observed.

Close
