# Supplementary material for: Draft genome assemblies using sequencing reads from Oxford Nanopore Technology and Illumina platforms for four species of North American Fundulus killifish
Source: Gigascience. 2020 Jun 18;9(6):giaa067. doi: 10.1093/gigascience/giaa067 (PMC7301629; doi:10.1093/gigascience/giaa067)
Supplement: giaa067_Reviewer_1_Report_Original_Submission — Chao Bian -- 10/30/2019 Reviewed [file giaa067_reviewer_1_report_original_submission.pdf]

Reviewer Report

Title: Draft genome assemblies using sequencing reads from Oxford Nanopore Technology and Illumina platforms for four species of North American Fundulus killifish

Version: Original Submission **Date:** 10/30/2019

Reviewer name: Chao Bian

Reviewer Comments to Author:

The authors from this paper "Draft genome assemblies using sequencing reads from Oxford Nanopore Technology and Illumina platforms for four species of North American killifish from the Fundulus genus" has reported four species of famous Fundulus killifish assembled by the combination of ONT data and Illumina data. The quality of these four assemblies was pretty well. Their BUSCO results also showed high completeness. The assembling pipeline was described in detail.
However, the language of this article should be gone through carefully. Too much errors were in the manuscript. As well as, only assembly data were really limited.
For the abstract: the format should be revised for gigascience publication.
Key words: "genome assembly" should be genome assemblies.
Lines 32-35, this sentence is too heavy, please split it to be clearer.
Line 56, remove a "genome" from "genome genome assembly".
The decimal place of number should be uniform through whole article, 91.2 Gb in line 50, 110.96 Gb in line 52 and 70.6 Gb in 63 should be changed.
Line 72, "after polishing" should be changed to "after being polished".
Line 77, how about "repeated genomic divergence". It means multiple genomic divergence?
Line 91, what's the real meaning of "the functional basis of, and evolution of,"?
For the Figure 1, the quality of this figure is too poor, please revise.
Line 128, for the "both the ultra-long read sequencing protocol from [42]", "from" what?
Line 142, "tissue" should tissues.
Line 150, "reads N50" should be changed to "read N50".
Only the genome assemblies are indeed limited for further using, like comparative genomic analysis. This will seriously impede the power of this important article and these famous species. I strongly suggest the authors to perform the genome annotation of these four killifish, and re-submit this article.

Level of Interest

Please indicate how interesting you found the manuscript: Choose an item.

Quality of Written English

Please indicate the quality of language in the manuscript: Choose an item.

Declaration of Competing Interests

Please complete a declaration of competing interests, considering the following questions:

- Have you in the past five years received reimbursements, fees, funding, or salary from an organisation that may in any way gain or lose financially from the publication of this manuscript, either now or in the future?
- Do you hold any stocks or shares in an organisation that may in any way gain or lose financially from the publication of this manuscript, either now or in the future?
- Do you hold or are you currently applying for any patents relating to the content of the manuscript?
- Have you received reimbursements, fees, funding, or salary from an organization that holds or has applied for patents relating to the content of the manuscript?
- Do you have any other financial competing interests?
- Do you have any non-financial competing interests in relation to this paper?

If you can answer no to all of the above, write 'I declare that I have no competing interests' below. If your reply is yes to any, please give details below.

I declare that I have no competing interests

I agree to the open peer review policy of the journal. I understand that my name will be included on my report to the authors and, if the manuscript is accepted for publication, my named report including any attachments I upload will be posted on the website along with the authors' responses. I agree for my report to be made available under an Open Access Creative Commons CC-BY license (http://creativecommons.org/licenses/by/4.0/). I understand that any comments which I do not wish to be included in my named report can be included as confidential comments to the editors, which will not be published.

Choose an item.

To further support our reviewers, we have joined with Publons, where you can gain additional credit to further highlight your hard work (see: https://publons.com/journal/530/gigascience). On publication of this paper, your review will be automatically added to Publons, you can then choose whether or not to claim your Publons credit. I understand this statement.

Yes Choose an item.
