# Supplementary material for: Draft genome assemblies using sequencing reads from Oxford Nanopore Technology and Illumina platforms for four species of North American Fundulus killifish
Source: Gigascience. 2020 Jun 18;9(6):giaa067. doi: 10.1093/gigascience/giaa067 (PMC7301629; doi:10.1093/gigascience/giaa067)
Supplement: giaa067_Reviewer_2_Report_Original_Submission — Jason R. Miller, MS -- 10/30/2019 Reviewed [file giaa067_reviewer_2_report_original_submission.pdf]

Reviewer Report

Title: Draft genome assemblies using sequencing reads from Oxford Nanopore Technology and Illumina platforms for four species of North American Fundulus killifish

Version: Original Submission **Date:** 10/30/2019

Reviewer name: Jason R. Miller, MS

Reviewer Comments to Author:

Summary of the Paper
The authors report the availability of raw sequencing reads plus genome assemblies for four fish genomes of one genus. The introduction explains the potential value of these four genomes for evolutionary studies. For each species, genome assemblies were generated wtdbg2 on ONT data followed by pilon polishing using Illumina data. Illumina-only and ONT-only assemblies are mentioned for comparison. Assembly quality was assessed with contig size statistics and BUSCO gene counts. The data is accessible online.
As is appropriate for a Data Note, the manuscript lacks the analysis that is expected of a genomes paper. It does not present coverage analysis, which could indicate areas of mis-assembly. It does not include the gene annotation of any assembly. It does not include any alignments between the four new assemblies or the previously published one. It does not speculate on possible causes of the six-fold variation in contig N50.
Minor Revisions Requested
1. The tables and figures could use some adjustment to increase their value to readers. The caption to Table 1 should explain or reference the N50 vs n statistics in column 5, and the Q>5 threshold in columns 6 and 7. The caption to Figure 3 should label the five colored wiggle plots that appear in 3A. Table 2 should use significant digits consistently. Table 3 should list the species in the same order as the other tables for ease of comparison.
2. The discussion of cost should be more disciplined. This paper refers to "the low cost" of its assemblies in contrast to "high cost of generation" with other technologies. However, no cost data is presented or referenced. Prices are difficult to pin down but there must be some way to provide a helpful comparison backed by facts.
3. The title names two companies. Could it instead refer to polished nanopore assemblies?
4. The discussion section should be revised to remove imprecise wording. Quality standards of the VGP could use better explanation and citation. The phrase "these assemblies" is ambiguous in places. Pilon "served to correct errors"; which kind? The "qualities of the ONT data appeared to make a difference"; which qualities? "It might matter more to have lower yield of longer reads"; matter how? Anyway, is that a setting on the sequencer? "Our samples were consistently not using pores as efficiently" seems to be a colloquialism. A "brittle property" of the DNA is probably speculation. One paper's recommendation for synteny analysis may not warrant being called THE minimum requirements.

Level of Interest

Please indicate how interesting you found the manuscript: Choose an item.

Quality of Written English

Please indicate the quality of language in the manuscript: Choose an item.

Declaration of Competing Interests

Please complete a declaration of competing interests, considering the following questions:

- Have you in the past five years received reimbursements, fees, funding, or salary from an organisation that may in any way gain or lose financially from the publication of this manuscript, either now or in the future?
- Do you hold any stocks or shares in an organisation that may in any way gain or lose financially from the publication of this manuscript, either now or in the future?
- Do you hold or are you currently applying for any patents relating to the content of the manuscript?
- Have you received reimbursements, fees, funding, or salary from an organization that holds or has applied for patents relating to the content of the manuscript?
- Do you have any other financial competing interests?
- Do you have any non-financial competing interests in relation to this paper?

If you can answer no to all of the above, write 'I declare that I have no competing interests' below. If your reply is yes to any, please give details below.

I declare that I have no competing interests

I agree to the open peer review policy of the journal. I understand that my name will be included on my report to the authors and, if the manuscript is accepted for publication, my named report including any attachments I upload will be posted on the website along with the authors' responses. I agree for my report to be made available under an Open Access Creative Commons CC-BY license (http://creativecommons.org/licenses/by/4.0/). I understand that any comments which I do not wish to be included in my named report can be included as confidential comments to the editors, which will not be published.

Choose an item.

To further support our reviewers, we have joined with Publons, where you can gain additional credit to further highlight your hard work (see: https://publons.com/journal/530/gigascience). On publication of this paper, your review will be automatically added to Publons, you can then choose whether or not to claim your Publons credit. I understand this statement.

Yes Choose an item.
