# Supplementary material for: Draft genome assemblies using sequencing reads from Oxford Nanopore Technology and Illumina platforms for four species of North American Fundulus killifish
Source: Gigascience. 2020 Jun 18;9(6):giaa067. doi: 10.1093/gigascience/giaa067 (PMC7301629; doi:10.1093/gigascience/giaa067)
Supplement: giaa067_Reviewer_3_Report_Original_Submission — Christiaan Henkel -- 10/31/2019 Reviewed [file giaa067_reviewer_3_report_original_submission.pdf]

Reviewer Report

Title: Draft genome assemblies using sequencing reads from Oxford Nanopore Technology and Illumina platforms for four species of North American Fundulus killifish

Version: Original Submission **Date:** 10/31/2019

Reviewer name: Christiaan Henkel

Reviewer Comments to Author:

This is a useful study describing the sequencing and initial assembly of four Fundulus species. Although there is a lot of interesting genome biology to be discovered, this paper focuses on the technicalities of data collection only. To be useful for the intended benchmarking purposes, I would like to ask the authors to supply some additional details on several stages of the sequencing and analyses:
P3, Methods and Results: How did you identify the species?
P5, ONT sequencing: I assume you used R9.4(.1?) flowcells. Please list the exact types, as these have changed several times already and are about to change again. Also, which version of which ONT basecaller did you use?
P6, Table 1: Coverage is calculated using a reference species genome, but it should be straightforward to estimate it for each species based on Illumina k-mer profiles. The assembly sizes (Tables 3,4) vary by ~10%, it would be nice to know whether this reflects actual differences in genome size between the species. In addition, this will yield an estimate of heterozygosity, which is relevant for the interpretation of the Illumina assemblies (high heterozygosity may result in harder to assemble genomes when using short reads).
P8/9, BUSCO scores: It would be very informative if you included a positive control here, by running BUSCO on the F. heteroclitus reference genome. (PS I am somewhat surprised the fragmented Illumina assemblies yield relatively decent completeness scores. Could this be a result of the specific reference database (eukaryotes) used? Perhaps strongly conserved genes are often found in regions of the genome that are relatively easy to assemble?)
P9, l221: How did you run Pilon? Some recommend running it multiple times? Polishing will have changed the genome lengths/N50s listed in Table 4.
P10, Discussion: There is some variation in sample storage conditions, DNA isolation, library prep methods, etc. As far as I can see, there are no clear correlations with data and assembly quality, but perhaps you could summarize this here?
Some minor thoughts & comments:
P2, l49: Although PromethION has higher throughput than MinION, this section also compares ONT data from different timepoints. Changes in chemistry and protocols are probably of greater influence on the increases in throughput than the platform. Today, MinION throughput is (under good conditions) higher than the 6.5 Gbp mentioned.
P2, l67, 'Recently…': This approach using Illumina polishing is not of a more recent date than the scaffolding method. Fish genomes produced using either method were published in 2017 already.
P3, l89: This sentence introduces the Atlantic killifish twice.

P9, l220: I assume the 6.1 hours for wtdbg2 are not CPU hours (as listed for Pilon, next line)? I have used it to assemble a similar genome, which took ~350 CPU hours.
P10, Discussion, third paragraph: DNA from fatty fish is notoriously difficult to sequence, with low yields due to rapid pore deaths. I think the cause remains unknown (and the phenomenon unpublished?). Although the fish sequenced here are not that rich in oils, did you observe the same phenomenon (i.e. when you mention 'not using pores as efficiently')?

Level of Interest

Please indicate how interesting you found the manuscript: Choose an item.

Quality of Written English

Please indicate the quality of language in the manuscript: Choose an item.

Declaration of Competing Interests

Please complete a declaration of competing interests, considering the following questions:

- Have you in the past five years received reimbursements, fees, funding, or salary from an organisation that may in any way gain or lose financially from the publication of this manuscript, either now or in the future?
- Do you hold any stocks or shares in an organisation that may in any way gain or lose financially from the publication of this manuscript, either now or in the future?
- Do you hold or are you currently applying for any patents relating to the content of the manuscript?
- Have you received reimbursements, fees, funding, or salary from an organization that holds or has applied for patents relating to the content of the manuscript?
- Do you have any other financial competing interests?
- Do you have any non-financial competing interests in relation to this paper?

If you can answer no to all of the above, write 'I declare that I have no competing interests' below. If your reply is yes to any, please give details below.

I have received travel reimbursements from Oxford Nanopore Technologies.

I agree to the open peer review policy of the journal. I understand that my name will be included on my report to the authors and, if the manuscript is accepted for publication, my named report including any attachments I upload will be posted on the website along with the authors' responses. I agree for my report to be made available under an Open Access Creative Commons CC-BY license (http://creativecommons.org/licenses/by/4.0/). I understand that any comments which I do not wish to

be included in my named report can be included as confidential comments to the editors, which will not be published.

Choose an item.

To further support our reviewers, we have joined with Publons, where you can gain additional credit to further highlight your hard work (see: https://publons.com/journal/530/gigascience). On publication of this paper, your review will be automatically added to Publons, you can then choose whether or not to claim your Publons credit. I understand this statement.

Yes Choose an item.
